# Secondary amine-initiated three-component synthesis of 3,4-dihydropyrimidinones and thiones involving alkynes, aldehydes and thiourea/urea

**DOI:** 10.3762/bjoc.10.25

**Published:** 2014-01-29

**Authors:** Jie-Ping Wan, Yunfang Lin, Kaikai Hu, Yunyun Liu

**Affiliations:** 1 Key Laboratory of Functional Small Organic Molecules, Ministry of Education and College of Chemistry and Chemical Engineering, Jiangxi Normal University, Nanchang 330022, P. R. China

**Keywords:** alkynes, DHPMs, diversity, enamine activation, multicomponent reactions

## Abstract

The three-component reactions of aldehydes, electron deficient alkynes and ureas/thioureas have been smoothly performed to yield a class of unprecedented 3,4-dihydropyrimidinones and thiones (DHPMs). The reactions are initiated by the key transformation of an enamine-type activation involving the addition of a secondary amine to an alkyne, which enables the subsequent incorporation of aldehydes and ureas/thioureas. This protocol tolerates a broad range of aryl- or alkylaldehydes, *N*-substituted and unsubstituted ureas/thioureas and alkynes to yield the corresponding DHPMs with specific regioselectivity.

## Introduction

**Figure 1:**
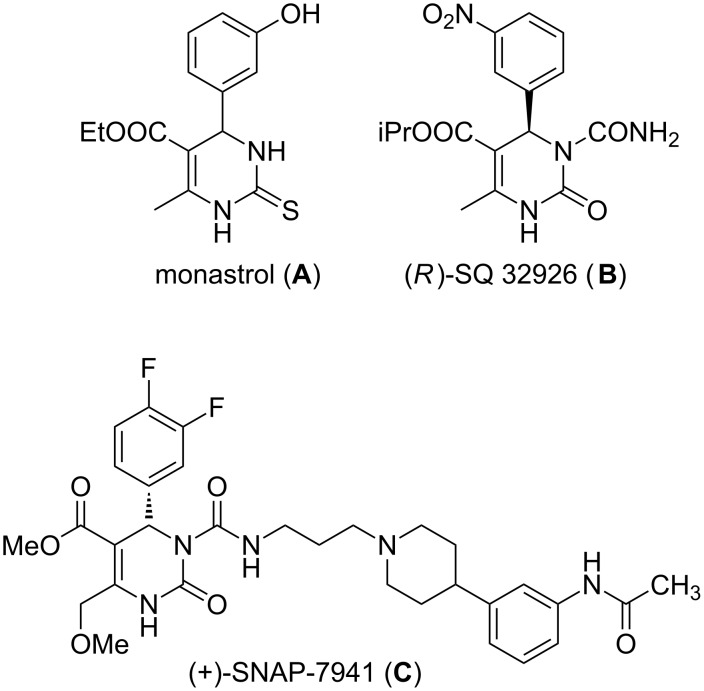
Some DHPMs-based lead compounds.

DHPMs are well-known heterocyclic scaffolds with abundant biological relevance [1–3]. The DHPM backbone has been found in a class of marine natural products possessing anti-HIV activity [4]. What’s more, diversified other biological activities have been discovered in many synthesized small DHPMs. For example, monastrol (**A**) [5], (R)-SQ 32926 (**B**) [6] and (+)-SNAP-7941(**C**) [7] are lead compounds possessing outstanding antitumor, antihypertensive and melanin-concentrating hormone receptor antagonism activities, respectively (Figure 1).

More recently, it was shown that DHPMs display many new bioactivities such as antioxidation [8], antibacterial [9], antimalaria [10], antimicrobial [11] and sodium iodide symporter inhibition [12], suggesting the great potential of DHPMs in discovering new lead compounds and medicines. Besides their attractiveness in biological and medicinal researches, DHPMs have also been demonstrated as quite flexible precursors for the synthesis of many other derived heterocyclic scaffolds [13].

**Scheme 1:**
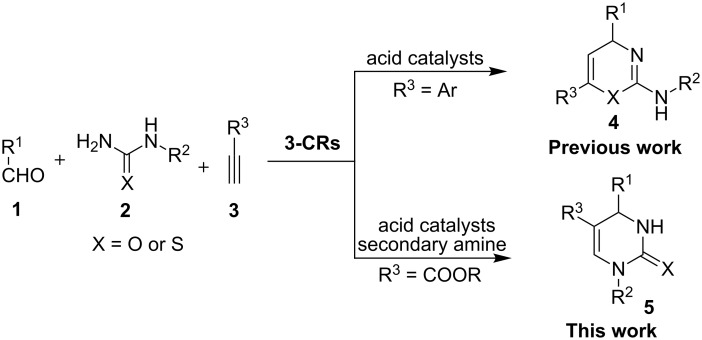
Regioselective 1,3-thiazines and DHPMs via aldehydes, ureas/thioureas and alkynes.

For a rather long period, the Biginelli reaction involving the condensation of aldehydes, β-ketoesters and ureas (thioureas) [14] has been dominantly employed for DHPMs synthesis in both racemic [15–18] and asymmetric versions [19–23]. Despite of many recognized advantages of the Biginelli reaction, the product diversity suffered from limitations because β-ketoesters or 1,3-diketones are intrinsically required as donors of the C5–C6 fragment in this reaction, which predetermined the presence of a C6 substitution in the produced DHPMs. On the other hand, DHPMs without a substituent at the C6 site were hardly accessible by the classical Biginelli reaction, probably because of either the rare availability of the corresponding β-formylketone/ester substrates or the intolerance of β-formylketones/esters in the Biginelli reaction. In regard to the daily increasing requirements on molecular diversity, developing powerful methods for the rapid synthesis of DHPMs with diverse and unprecedented substitution patterns has become an issue of central importance. During the last decade, tremendous endeavours have been made to devise efficient synthetic routes to access structurally diverse DHPMs by employing multicomponent reactions (MCRs) [24–26]. Interestingly, in the process of designing new MCRs yielding DHPMs, the utilization of new C5–C6 fragment donors constituted the major strategy. Representative new C5–C6 building blocks used in the multicomponent synthesis of DHPMs are 2-oxosuccinic acid [27], acetylaldehydes [28], cyclic and acyclic ketones [29–31], β-oxo dithioesters [32], diketenes [33] and enaminones [34]. On the other hand, as frequently utilized building blocks in organic synthesis, alkynes have been known to possess versatile reactivity in the synthesis of small molecules. For example, a previous protocol employing aryl alkynes, aldehydes and urea/thiourea has been found to selectively provide various 1,3-thiazine derivatives **4** [35]. Amazingly, a generally applicable alkyne-based method regioselectively yielding DHPMs has not yet been achieved [36]. Herein, we report the regioselective three-component synthesis of DHPMs employing alkynes, aldehydes and ureas/thioureas by making use of the activation effect of a secondary amine to alkynes (Scheme 1) [37].

## Results and Discussion

The work began from the three-component model reaction of *p*-chlorobenzaldehyde (**1a**), thiourea (**2a**) and ethyl propiolate (**3a**). The optimization results are outlined in Table 1. Firstly, parallel studies respectively employing TMSCl, morpholine and mixed TMSCl/morpholine as catalysts have been conducted. It was found that the target product could be formed only when both morpholine and TMSCl were present (Table 1, entries 1–3). Extended experiments using different amounts and types of amine catalysts demonstrated that 0.5 equiv of piperazine was favorable (Table 1, entries 4–6). Reducing the amount of TMSCl led to a decrease in product yield (Table 1, entry 7). Other Lewis acid or Brønsted acids such as FeCl_3_ and *p*-TSA gave no better result for the same reaction (Table 1, entries 8 and 9). In addition, the non-polar solvent toluene was not able to mediate the reaction, while a lower yield of product was observed when MeCN was used as solvent (Table 1, entries 10 and 11). Altering the reaction temperature also failed to enhance the yield (Table 1, entries 12 and 13). Finally, employing additional 0.5 equiv of *p*-TSA has been found to significantly improve the yield (Table 1, entry 14). This result may be attributed to the double activation effect involving both Lewis and Brønsted acid (see Scheme 3).

**Table 1:** Optimization of reaction conditions^a^.

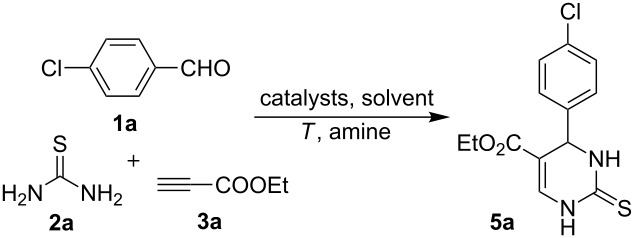				

Entry	Catalysts	Solvent	*T* (°C)	Yield (%)^b^

1	morpholine/TMSCl	DMF	90	45
2^c^	TMSCl	DMF	90	nr
3^c^	morpholine	DMF	90	nr
4^d^	morpholine/TMSCl	DMF	90	25
5	pyrrolidine/TMSCl	DMF	90	13
6	piperazine/TMSCl	DMF	90	59
7^e^	piperazine/TMSCl	DMF	90	40
8	piperazine/FeCl_3_	DMF	90	nr
9	piperazine/*p*-TSA	DMF	90	15
10	piperazine/TMSCl	CH_3_CN	90	39
11	piperazine/TMSCl	toluene	90	nr
12	piperazine/TMSCl	DMF	80	27
13	piperazine/TMSCl	DMF	100	39
14^f^	piperazine/TMSCl	DMF	90	81

^a^General conditions: **1a** (0.3 mmol), **2a** (0.4 mmol), **3a** (0.3 mmol), secondary amine (0.15 mmol) and acid (0.6 mmol) in 4 mL solvent, stirred for 12 h. ^b^Yields of isolated product. ^c^No reaction. ^d^0.09 mmol (30 mol %) morpholine was used. ^e^0.45 mmol TMSCl was used. ^f^Additional 0.5 equiv of *p*-TSA was used.

With the optimal conditions in hand, we conducted the investigation on examining the application scope. Various aldehydes of different properties have been subjected to react with thioureas/*N*-substituted thioureas/urea as well as different propiolates. Typical results were listed in Table 2. It can be seen from these reactions that aldehydes containing various functional groups tolerate the protocol of the corresponding DHPMs synthesis. For reactions involving aromatic aldehydes, the electronic properties of the substituent exhibited evident impact on the product yield. Aldehydes containing an electron withdrawing group (EWG) facilitated the reactions to give related DHPMs with evidently higher yields than those containing an electron donating group (EDG) (Table 2, products **5a**–**5e**, **5i**–**5k**). A similar tendency occurred in the experiments using *N*-methyl thiourea (Table 2, products **5f**–**5h**). Attempts on employing EDG-substituted aldehydes such as *p*-tolylaldehyde to react with *N*-substituted thiourea and alkyne were not successful. On the other hand, benzaldehydes with *ortho*- and *meta*-substitution could also react with thioureas and propiolates to give the corresponding DHPMs **5l**–**5p**. However, compared with thiourea, urea has been found to undergo a similar transformation more toughly, and DHPMs **5q–5s** from urea reactions have been obtained with only moderate yields under the conditions of refluxing THF (Table 2, products **5q**–**5s**). Notably, this synthetic methodology displayed also good tolerance to aliphatic aldehydes to provide 4-alkyl DHPMs **5t**–**5v** with good to excellent yield (Table 2).

**Table 2:** Multicomponent synthesis of different DHPMs.^a^

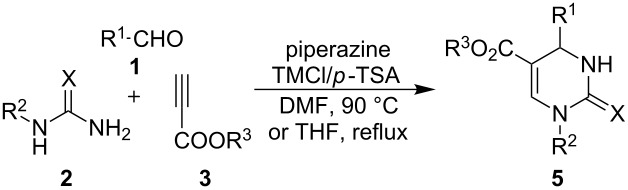					

R^1^	R^2^	R^3^	X	Product	Yield (%)^b^

4-ClC_6_H_4_	H	Et	S	**5a**	81
4-BrC_6_H_4_	H	Et	S	**5b**	70
4-CF_3_C_6_H_4_	H	Et	S	**5c**	72
4-NO_2_C_6_H_4_	H	Et	S	**5d**	85
4-MeC_6_H_4_	H	Et	S	**5e**	58
4-ClC_6_H_4_	Me	Et	S	**5f**	78
4-BrC_6_H_4_	Me	Et	S	**5g**	63
4-CF_3_C_6_H_4_	Me	Et	S	**5h**	83
4-ClC_6_H_4_	H	Me	S	**5i**	72
4-CF_3_C_6_H_4_	H	Me	S	**5j**	81
4-MeC_6_H_4_	H	Me	S	**5k**	66
3-OHCC_6_H_4_	H	Et	S	**5l**	68
3-MeOC_6_H_4_	H	Et	S	**5m**	61
2,4-Cl_2_C_6_H_3_	H	Et	S	**5n**	64
2-ClC_6_H_4_	Me	Et	S	**5o**	75
2-ClC_6_H_4_	H	Me	S	**5p**	60
4-ClC_6_H_4_	H	Et	O	**5q**^c^	43
4-BrC_6_H_4_	H	Et	O	**5r**^c^	55
4-NO_2_C_6_H_4_	H	Et	O	**5s**^c^	47
Et	H	Et	S	**5t**	82
Pr	H	Et	S	**5u**	68
PhCH_2_	H	Et	S	**5v**	81

^a^General conditions: **1** (0.3 mmol), **2** (0.4 mmol), **3** (0.3 mmol), piperazine (0.15 mmol), TMSCl (0.6 mmol), *p*-TSA (0.15 mmol) in 4 mL DMF, stirred at 90 °C for 12 h. ^b^Yield of isolated product. ^c^Reactions in refluxing THF, piperazine (0.15 mmol), TMSCl (0.9 mmol) and *p*-TSA (0.3 mmol).

**Scheme 2:**
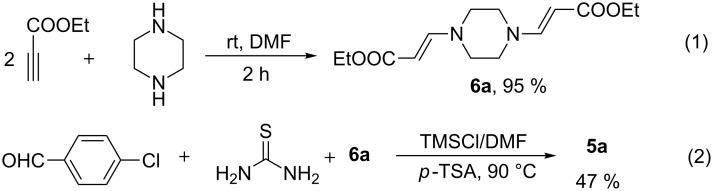
Synthesis of enamino ester intermediate and its transformation to DHPM.

Following these obtained results, especially the key function of the secondary amine to activate electron deficient alkynes [34] we conducted the control experiments on both the synthesis of the possible enamino ester and its transformation to the corresponding DHPM product. The results proved that enamino ester **6a** could be easily generated and efficiently transformed to target product **5a** under standard conditions (without using secondary amine, Scheme 2).

**Scheme 3:**
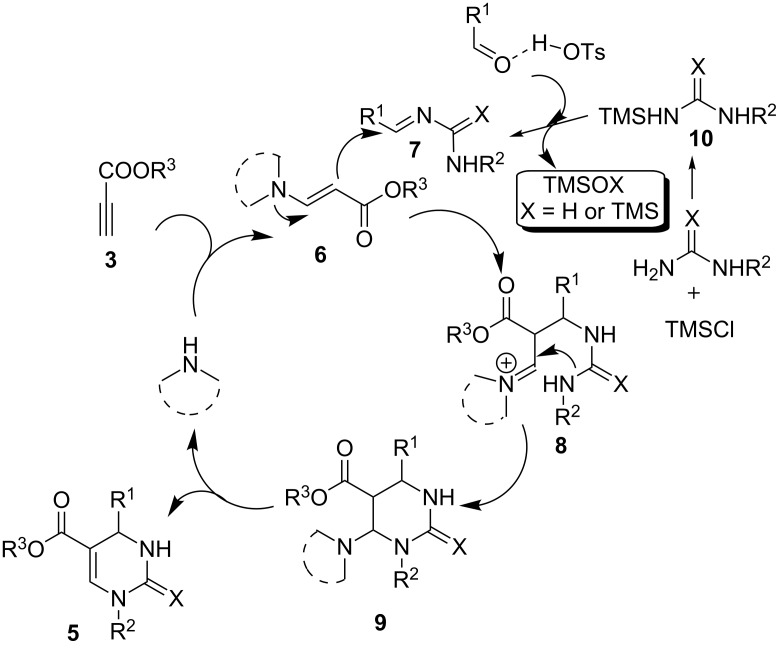
Proposed reaction mechanism.

Based on the results from the control experiments, we postulate the reaction mechanism: At first, the addition of the secondary amine to the propiolate gives enamino ester intermediate **6**. On the other hand, ureas/thioureas were known to be readily activated by TMSCl to give intermediate **10** [38–39]. Intermediate **10** consequently condenses with the aldehyde which was activated by *p*-TSA to generate imine **7**. The combination of **6** and **7** allows the production of iminium ion **8**. Finally, an intramolecular cyclization of **8** leads to the formation of **9** which subsequently undergoes deaminative elimination to result product **5** by releasing the amine catalyst for further recycling (Scheme 3).

## Conclusion

In conclusion, we have established an unprecedented amine-initiated three-component protocol for the synthesis of new DHPMs wherein readily available alkynes served as C5–C6 building blocks. This methodology displayed general applicability for aryl- and alkylaldehydes, urea, thiourea, *N*-substituted thiourea and different alkyl propiolates. The method is useful for the synthesis of diverse new DHPMs which were hardly accessible through known methods such as the Biginelli reaction.

## Experimental

### General information

All reagents were obtained from commercial sources and used directly without further purification, solvents have been treated following standard processes prior to use. ^1^H and ^13^C NMR spectra were recorded on a 400 MHz or 600 MHz apparatus. The frequencies for ^1^H NMR and ^13^C NMR experiments are 400 MHz/600 MHz and 100 MHz/150 MHz, respectively. The chemical shifts were reported in ppm employing TMS as internal standard. Melting points were measured with an X-4A instrument without correcting the temperature, IR spectra were measured in KBr on a Spectrum One apparatus and the HRMS were obtained under ESI mode in a Bruker 7-tesla FT-ICR MS instrument.

### General procedure for the three-component synthesis of DHPMs **5**

Aldehyde **1** (0.3 mmol), urea/thiourea **2** (0.4 mmol) and alkyl propionate **3** (0.3 mmol) piperazine (0.15 mmol), and *p*-tolylsulfonic acid (0.15 mmol, 0.3 mmol for the reaction of urea) were charged in a 25 mL round bottom flask equipped with a stirring bar. DMF (THF for the reaction of urea) (4 mL) and TMSCl (0.6 mmol, 0.9 mmol for the reaction of urea) were added and the mixture was stirred at 90 °C for 12 h (TLC). After cooling down to room temperature, 5 mL water was added, and the resulting mixture was extracted with ethyl acetate (3 × 8 mL). The organic layers were combined and dried overnight with anhydrous MgSO_4_. After filtration and removing of the solvent under reduced pressure, the residue was subjected to flash column chromatography to provide pure products.

**Synthesis of intermediate 6a.** Into a 25 mL round bottom flask was added ethyl propiolate (0.6 mmol) and piperazine (0.3 mmol). 1.5 mL DMF was added and the mixture was stirred at rt for 8 h (TLC). Upon completion, 10 mL water was added and the resulting mixture was extracted with EtOAc (3 × 10 mL). The combined organic layer was dried with anhydrous Na_2_SO_4._ After removing of the solid by filtration and evaporation of the solvent the product **6a** was isolated as white solid.

## Supporting Information

File 1 Experimental details on the synthesis of all DHPMs **5** and intermediate **6a**, full characterization data as well as ^1^H and ^13^C NMR spectra of all products **5** and **6a**.
