# Supplementary material for: Secondary amine-initiated three-component synthesis of 3,4-dihydropyrimidinones and thiones involving alkynes, aldehydes and thiourea/urea
Source: Beilstein J Org Chem. 2014 Jan 29;10:287–92. doi: 10.3762/bjoc.10.25 (PMC3943560; doi:10.3762/bjoc.10.25)
Supplement: File 1 — Experimental details on the synthesis of all DHPMs 5 and intermediate 6a, full characterization data as well as 1H and 13C NMR spectra of all products 5 and 6a. [file Beilstein_J_Org_Chem-10-287-s001.pdf]

Supporting Information

for

Secondary amine-initiated three-component synthesis of 3,4-dihydropyrimidinones and thiones involving alkynes, aldehydes and thiourea/urea

Jie-Ping Wan*, Yunfang Lin, Kaikai Hu, Yunyun Liu

Address: Key Laboratory of Functional Small Organic Molecules, Ministry of Education and College of Chemistry and Chemical Engineering, Jiangxi Normal University, Nanchang 330022, P. R. China

Email: Jie-Ping Wan - wanjieping@gmail.com

* Corresponding author

Experimental details on the synthesis of all DHPMs 5 and intermediate 6a, full characterization data as well as ^{1}H and ^{13}C NMR spectra of all products 5 and 6a.

Contents

Characterization data of products & intermediate…………………….…S2–S13

^{1}H and ^{13}C NMR spectra of products & intermediate…….…………...…S14–S36

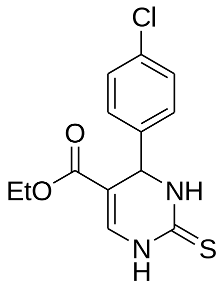

Ethyl 4-(4-chlorophenyl)-2-thioxo-1,2,3,4-tetrahydropyrimidine-5-carboxylate (5a). Pale yellow solid, m.p.: 238-239 °C; ^{1}H NMR (400MHz, DMSO-d_6) δ = 10.46 (d, 1 H, *J* = 4.8 Hz, HN), 9.63 (s, 1 H, NH), 7.44 (d, 2 H, *J* = 8.4 Hz, ArH), 7.26 (d, 2 H, *J* = 8.4 Hz, ArH), 7.15 (d, 1 H, *J* = 7.2 Hz, CH=), 5.16 (d, 1 H, *J* = 3.2 Hz, CH), 4.06-4.00 (m, 2 H, OCH_2), 1.13 (t, 3 H, *J* = 6.8 Hz, CH_3 in Et); ^{13}C NMR (100 MHz, DMSO-d_6) δ = 174.09, 164.32, 141.97, 132.98, 132.45, 128.59, 128.47, 104.30, 59.87, 53.24, 13.98; IR (KBr, cm^{-1}): 3282, 3179, 1700, 1679, 1656, 1195; ESI-HRMS: Calcd for $C_{13}H_{14}ClN_2O_2S[M+H]^+$: 297.0465; Found: 297.0462.

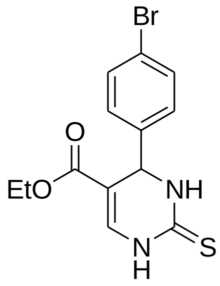

Ethyl 4-(4-bromophenyl)-2-thioxo-1,2,3,4-tetrahydropyrimidine-5-carboxylate (5b). White solid, m.p.: 244-246 °C; ^{1}H NMR (400MHz, DMSO-d_6) δ = 10.47 (d, 1 H, *J* = 4.0 Hz, NH), 9.64 (s, 1 H, NH), 7.58 (d, 2 H, *J* = 7.6 Hz, ArH), 7.19 (d, 2 H, *J* = 7.2 Hz, ArH), 7.14 (d, 1 H, *J* = 4.8 Hz, CH=), 5.14 (s, 1 H, CH), 4.03 (q, 2 H, *J* = 7.2 Hz, OCH_2), 1.13 (t, 3 H, *J* = 7.2 Hz, CH_3 in Et); ^{13}C NMR (100 MHz, DMSO-d_6) δ = 174.25, 164.34, 142.40, 133.01, 131.50, 128.79, 120.98, 104.36, 59.86, 53.38,

13.98; IR (KBr, cm^{-1}): 3262, 3188, 1703, 1673, 1649, 1192; ESI-HRMS: Calcd for $C_{13}H_{14}BrN_2O_2S[M+H]^+$: 340.9959; Found: 340.9947.

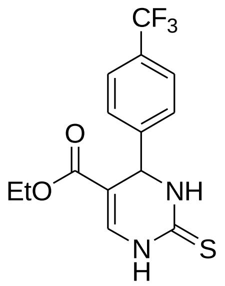

Ethyl 2-thioxo-4-(4-(trifluoromethyl)phenyl)-1,2,3,4-tetrahydropyrimidine-5-carboxylate (5c). Pale yellow solid, m.p.: 222-223 °C; ^{1}H NMR (400MHz, DMSO-d_6) δ = 10.45 (brs, 1 H, NH), 9.62 (s, 1 H, NH), 7.76 (d, 2 H, J = 7.6 Hz, ArH), 7.46 (q, 2 H, J = 7.6Hz, ArH), 7.22 (d, 1 H, J = 5.6 Hz, CH=), 5.32 (s, 1 H, CH), 4.07 (q, 2 H, J = 8.0 Hz, OCH_2), 1.15 (t, 3 H, J = 7.2 Hz, CH_3 in Et); ^{13}C NMR (100 MHz, DMSO-d_6) δ = 174.39, 164.29, 147.34, 133.24, 128.50 (d, J_{C-F} = 31.0 Hz), 127.46, 125.56 (d, J_{C-F} = 3.0 Hz), 122.73, 104.09, 59.89, 53.59, 13.91; IR (KBr, cm^{-1}): 3285, 3184, 1703, 1678, 1656, 1172; ESI-HRMS: Calcd for $C_{14}H_{14}F_3N_2O_2S[M+H]^+$: 331.0728; Found: 331.0732.

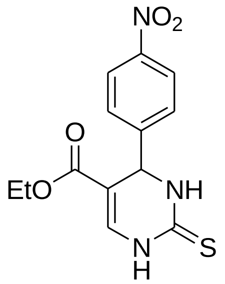

Ethyl 4-(4-nitrophenyl)-2-thioxo-1,2,3,4-tetrahydropyrimidine-5-carboxylate (5d). Yellow solid, m.p.: 106-109 °C; ^{1}H NMR (400MHz, DMSO-d_6) δ = 10.45 (d, 1 H, J = 4.0 Hz, NH), 9.61 (s,1 H, NH), 8.25 (d, 2 H, J = 8.0 Hz, ArH), 7.53 (d, 2 H, J = 8.4 Hz, ArH), 7.20 (d, 1 H, J = 5.6 Hz, CH=), 5.34 (s, 1 H, CH), 4.10-3.99 (m, 2 H,

OCH_2), 1.14 (t, 3 H, *J* = 7.2 Hz, CH_3 in Et); ^{13}C NMR (100 MHz, DMSO-d_6) δ = 174.94, 164.72, 150.37, 147.64, 133.92, 128.49, 124.37, 104.30, 60.46, 54.00, 14.45; IR (KBr, cm^{-1}): 3255, 3160, 1702, 1678, 1655, 1194; ESI-HRMS: Calcd for $C_{13}H_{14}N_3O_4S[M+H]^+$: 308.0705; Found: 308.0709.

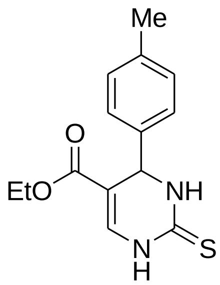

Ethyl 2-thioxo-4-*p*-tolyl-1,2,3,4-tetrahydropyrimidine-5-carboxylate (5e). Pale yellow solid, m.p.: 224-226 °C; ^{1}H NMR (400 MHz, DMSO-d_6) δ = 10.27 (brs, 1 H, NH), 9.48 (s, 1 H, NH), 7.15-7.12 (m, 5 H, ArH and CH=), 5.12 (s, 1 H, CH), 4.03 (q, 2 H, *J* = 7.2 Hz, OCH_2), 2.28 (s, 3 H, Ar-CH_3), 1.14 (t, 3 H, *J* = 6.8 Hz, CH_3 in Et); ^{13}C NMR (100 MHz, DMSO-d_6) δ = 174.12, 164.64, 140.19, 137.11, 132.65, 129.05, 126.42, 104.95, 59.76, 53.57, 20.60, 13.99; IR (KBr, cm^{-1}): 3289, 1682, 1654, 1191; ESI-HRMS: Calcd for $C_{14}H_{17}N_2O_2S[M+H]^+$: 277.1011; Found: 277.1012.

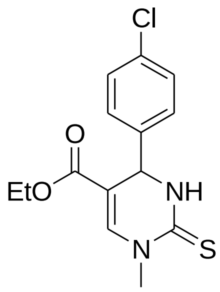

Ethyl 4-(4-chlorophenyl)-1-methyl-2-thioxo-1,2,3,4-tetrahydropyrimidine-5-carboxylate (5f). Pale yellow solid, m.p.: 168-170 °C; ^{1}H NMR (400MHz, DMSO-d_6) δ = 9.55 (s, 1 H, NH), 7.57 (s, 1 H, CH=), 7.42 (d, 2 H, *J* = 8.0 Hz, ArH), 7.27 (d, 2 H, *J* = 8.0 Hz, ArH), 5.17 (d, 1 H, *J* = 3.2 Hz, CH=), 4.09-4.03 (m, 2H,

OCH$_2$), 3.48 (s, 3 H, NCH$_3$), 1.15 (t, 3 H, J = 7.2 Hz, CH$_3$ in Et); ^{13}C NMR (100 MHz, DMSO-d_6) δ = 176.33, 164.22, 141.79, 137,97, 132.48, 128.55, 128.40, 105.71, 59.99, 53.07, 40.56, 14.02; IR (KBr, cm^{-1}): 3306, 1697, 1661, 1380, 1168; ESI-HRMS: Calcd for $C_{14}H_{16}ClN_2O_2S[M+H]^+$: 311.0621; Found: 311.0601.

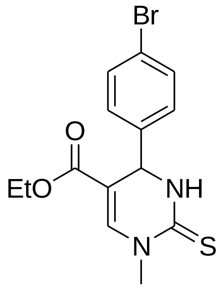

Ethyl 4-(4-bromophenyl)-1-methyl-2-thioxo-1,2,3,4-tetrahydropyrimidine-5-carboxylate (5g). Pale yellow solid, m.p.: 207-208 °C; ^{1}H NMR (400 MHz, DMSO-d_6) δ = 9.67 (s,1 H, NH), 7.59 (t, 3 H, J = 8.0 Hz, ArH and CH=), 7.21 (d, 2 H, J = 7.2 Hz, ArH), 5.14 (s, 1 H, CH), 4.07 (q, 2 H, J = 6.4 Hz, OCH$_2$), 3.48 (s, 3 H, NCH$_3$), 1.15 (t, 3 H, J = 6.4 Hz, CH$_3$ in Et); ^{13}C NMR (100 MHz, DMSO-d_6) δ = 176.32, 164.22, 142.20, 137.99, 131.49, 128.74, 121.00, 105.63, 59.99, 53.14, 40.56, 14.04; IR (KBr, cm^{-1}): 3302, 1693, 1667, 1383, 1167; ESI-HRMS: Calcd for $C_{14}H_{16}BrN_2O_2S[M+H]^+$: 355.0116; Found: 355.0122.

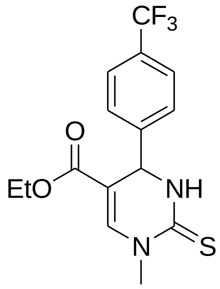

Ethyl 1-methyl-2-thioxo-4-(4-(trifluoromethyl)phenyl)-1,2,3,4-tetrahydropyrimidine-5-carboxylate (5h). Yellow solid, m.p.: 164-166 °C; ^{1}H NMR (400MHz, DMSO-d_6) δ = 9.62 (s,1 H, NH), 7.74 (d, 2 H, J = 8.0 Hz, ArH), 7.61 (s, 1 H, CH=),

7.48 (d, 2 H, *J* = 8.0 Hz, ArH), 5.27 (d, 1 H, *J* = 3.2 Hz, CH), 4.13-4.03 (m, 2 H, OCH_2), 3.50 (s, 3 H, NCH_3), 1.15 (t, 3 H, 7.2 Hz, CH_3 in Et); ^{13}C NMR (100 MHz, DMSO-d_6) δ = 176.48, 164.19, 147.15, 138.22, 128.48 (d, J_{C-F} = 31.0 Hz), 127.39, 125.56 (d, J_{C-F} = 3.0 Hz), 122.74, 105.37, 60.03, 53.34, 40.58, 13.98; IR (KBr, cm^{-1}): 3308, 1700, 1670, 1383, 1197; ESI-HRMS: Calcd for $C_{15}H_{16}F_3N_2O_2S[M+H]^+$: 345.0885; Found: 345.0862.

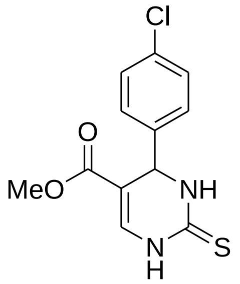

Methyl 4-(4-chlorophenyl)-2-thioxo-1,2,3,4-tetrahydropyrimidine-5-carboxylate (5i). White solid, m.p. 150-151 °C; ^{1}H NMR (400 MHz, DMSO-d_6) δ = 10.48 (d, 1 H, *J* = 4.8 Hz, NH), 9.66 (s, 1 H, NH), 7.45 (d, 2 H, *J* = 8.4 Hz, ArH), 7.26 (d, 2 H, *J* = 8.4 Hz, ArH), 7.17 (d, 1 H, *J* = 5.6 Hz, CH=), 5.16 (d, 1 H, *J* = 2.8 Hz, CH), 3.58 (s, 3H, OCH_3); ^{13}C NMR (100 MHz, DMSO-d_6) δ = 174.49, 165.29, 142.34, 133.74, 132.99, 129.16, 128.98, 104.43, 53.66, 51.86; IR (KBr, cm^{-1}): 3296, 1689, 1657, 1566, 1196; ESI-HRMS: Calcd for $C_{12}H_{12}ClN_2O_2S[M+H]^+$: 283.0308; Found: 283.0319.

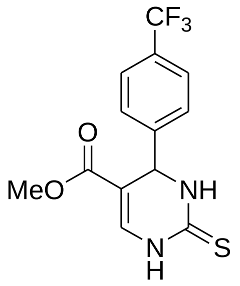

Methyl 2-thioxo-4-(4-(trifluoromethyl)phenyl)-1,2,3,4-tetrahydropyrimidine-5-carboxylate (5j). White solid, m.p.: 232-233 °C; ^{1}H NMR (400 MHz, DMSO-d_6) δ = 10.45 (brs,1 H, NH), 9.63 (s, 1 H, NH), 7.74 (d, 2 H, J = 7.6 Hz, ArH), 7.49 (d, 2 H, J = 7.2 Hz, ArH), 7.21 (d, 1 H, J = 5.2 Hz, CH=), 5.29 (s, 1 H, CH), 3.59 (s, 3 H, OCH_3); ^{13}C NMR (100 MHz, DMSO-d_6) δ = 174.88, 165.25, 147.74, 133.96, 129.01 (d, $J_{C\text{-}F}$ = 31.0 Hz), 127.95, 126.10 (d, $J_{C\text{-}F}$ = 3.0 Hz), 123.23, 104.27, 54.03, 51.78; IR (KBr, cm^{-1}): 3308, 1699, 1660, 1566, 1196; ESI-HRMS: Calcd for $C_{13}H_{12}F_3N_2O_2S[M+H]^+$: 317.0572; Found: 317.0580.

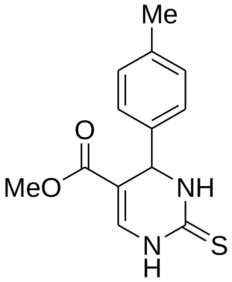

Methyl 2-thioxo-4-*p*-tolyl-1,2,3,4-tetrahydropyrimidine-5-carboxylate (5k). White solid, m.p.: 249-250 °C; ^{1}H NMR (400 MHz, DMSO-d_6) δ = 10.32 (brs,1H, NH), 9.54 (s, 1 H, NH), 7.16-7.12 (m, 5 H, ArH and CH=), 5.12 (d, 1 H, J = 2 Hz, CH), 3.59 (s, 3 H, OCH_3), 2.28 (s, 3 H, Ar-CH_3); ^{13}C NMR (100 MHz, DMSO-d_6) δ = 174.09, 164.91, 140.10, 137.14, 132.89, 129.09, 126.42, 104.61, 53.54, 51.20, 20.60; IR (KBr, cm^{-1}): 3299, 1687, 1654, 1567, 1191; ESI-HRMS: Calcd for $C_{13}H_{15}N_2O_2S[M+H]^+$: 263.0854; Found: 263.0848.

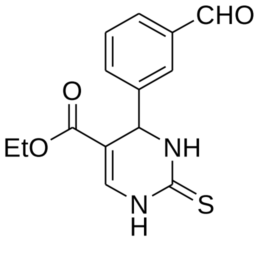

Ethyl 4-(3-formylphenyl)-2-thioxo-1,2,3,4-tetrahydropyrimidine-5-carboxylate (5l). Yellow solid, m.p.: 222-224 °C; ^{1}H NMR (400 MHz, DMSO-d_6) δ = 10.38 (brs, 1 H, CHO), 10.02 (s, 1 H, NH), 9.57(s, 1 H, NH), 7.87 (d, 1 H, J = 6.8 Hz, ArH), 7.77 (s, 1 H, ArH), 7.62 (q, 2 H, J = 7.6 Hz, ArH), 7.19 (d, 1 H, J = 5.2 Hz, CH=), 5.29 (s, 1 H, CH), 4.10 (q, 2 H, J = 7.6 Hz, OCH_2), 1.13 (t, 3 H, J = 6.8 Hz, CH_3 in Et); ^{13}C NMR (100 MHz, DMSO-d_6) δ = 192.83, 174.29, 164.32, 144.16, 136.53, 133.20, 132.74, 129.76, 129.57, 126.63, 104.26, 59.87, 53.69, 13.95; IR (KBr, cm^{-1}): 3275, 3181, 1695, 1659, 1565, 1194; ESI-HRMS: Calcd for $C_{14}H_{15}N_2O_3S[M+H]^+$: 291.0803; Found: 291.0822.

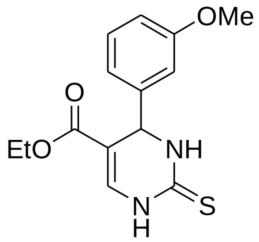

Ethyl 4-(3-methoxyphenyl)-2-thioxo-1,2,3,4-tetrahydropyrimidine-5-carboxylate (5m). White solid, m.p.: 200-202 °C; ^{1}H NMR (400 MHz, DMSO-d_6) δ = 10.44 (brs, 1 H, NH), 9.63 (s, 1 H, NH), 7.30 (q, 1 H, J = 7.6 Hz, ArH), 7.14 (t, 1 H, J = 5.6 Hz, CH=), 6.89-6.79 (m, 3 H, ArH), 5.13 (s, 1 H, CH), 4.05 (q, 2 H, J = 6.4 Hz, OCH_2), 3.74 (s, 3 H, OCH_3), 1.15 (t, 3 H, J = 6.8 Hz, CH_3 in Et); ^{13}C NMR (100 MHz, DMSO-d_6) δ = 174.62, 164.95, 159.77, 144.93, 133.38, 130.33, 118.96, 113.23, 113.12, 105.05, 60.38, 55.50, 54.06, 14.52; IR (KBr, cm^{-1}): 3281, 3181, 1693, 1679, 1565, 1192; ESI-HRMS: Calcd for $C_{14}H_{17}N_2O_3S[M+H]^+$: 293.0960; Found: 293.0954.

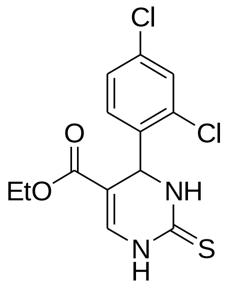

Ethyl 4-(2,4-dichlorophenyl)-2-thioxo-1,2,3,4-tetrahydropyrimidine-5-carboxylate (5n). Pale yellow solid, m.p.: 228-229 ^{o}C; ^{1}H NMR (400 MHz, DMSO-*d$_6$*) δ = 10.45 (brs, 1 H, NH), 9.57 (s, 1 H, NH), 7.62 (s, 1 H, ArH), 7.50 (d, 1 H, *J* = 7.6 Hz, ArH), 7.39 (d, 1 H, *J* = 8.4 Hz, ArH), 7.22 (d, 1 H, *J* = 5.6 Hz, CH=), 5.67 (d, 1 H, *J* = 2.0 Hz, CH), 4.06-3.98 (m, 2 H, OCH_2), 1.13 (t, 3 H, *J* = 7.2 Hz, CH_3 in Et); ^{13}C NMR (100 MHz, DMSO-*d$_6$*) δ = 173.71, 164.07, 139.29, 133.49, 133.19, 132.91, 131.42, 128.85, 127.99, 103.19, 59.83, 51.23, 13.91; IR (KBr, cm^{-1}): 3282, 3179, 1730, 1699, 1658, 1573, 1193; ESI-HRMS: Calcd for $C_{13}H_{13}Cl_2N_2O_2S[M+H]^+$: 331.0075; Found: 331.0074.

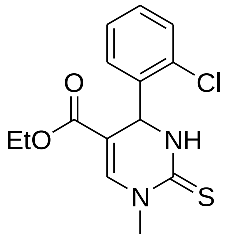

Ethyl 4-(2-chlorophenyl)-1-methyl-2-thioxo-1,2,3,4-tetrahydropyrimidine-5-carboxylate (5o). White solid, m.p.: 174-175 ^{o}C; ^{1}H NMR (400 MHz, DMSO-*d$_6$*) δ = 9.50 (s, 1 H, NH), 7.61 (s, 1 H, NH), 7.42 (d, 1 H, *J* = 7.2 Hz, ArH), 7.35-7.30 (m, 3 H, ArH and CH=), 5.63 (d, 1 H, *J* = 2.0 Hz, CH), 3.99 (q, 2 H, *J* = 6.8 Hz, OCH_2), 3.51 (s, 3 H, OCH_3), 1.08 (t, 3 H, *J* = 7.2 Hz, CH_3 in Et); ^{13}C NMR (100 MHz, DMSO-*d$_6$*) δ = 176.01, 164.04, 139.89, 138.37, 131.90, 129.88, 129.60, 129.52,

127.70, 105.02, 59.85, 51.53, 40.64, 13.92; IR (KBr, cm^{-1}): 3292, 1697, 1667, 1383, 1197; ESI-HRMS: Calcd for $C_{14}H_{16}ClN_2O_2S[M+H]^+$: 311.0621; Found: 311.0614.

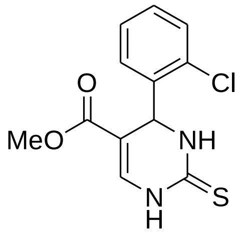

Methyl 4-(2-chlorophenyl)-2-thioxo-1,2,3,4-tetrahydropyrimidine-5-carboxylate (5p). Pale yellow solid, m.p.: 248-250 °C; ^{1}H NMR (400MHz, DMSO-d_6) δ = 10.47 (brs, 1 H, NH), 9.61 (s, 1 H, NH), 7.44-7.19 (m, 5 H, ArH and CH=), 5.63 (s, 1 H, CH), 3.53 (s, 3 H); ^{13}C NMR (100 MHz, DMSO-d_6) δ = 173.95, 164.58, 139.98, 133.54, 131.93, 129.84, 129.58, 127.72, 103.40, 51.66, 51.14; IR (KBr, cm^{-1}): 3282, 3169, 1703, 1678, 1656, 1193; ESI-HRMS: Calcd for $C_{12}H_{12}ClN_2O_2S[M+H]^+$: 283.0308; Found: 283.0300.

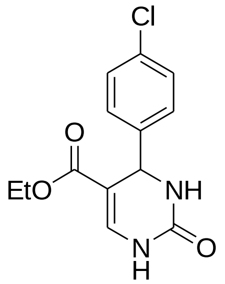

Ethyl 4-(4-chlorophenyl)-2-oxo-1,2,3,4-tetrahydropyrimidine-5-carboxylate (5q). Yellow solid, m.p.: 140-141 °C; ^{1}H NMR (600 MHz, DMSO-d_6) δ = 9.25 (d, 1 H, J = 6.0 Hz, NH), 7.71 (s, 1 H, NH), 7.39 (d, 2 H, J = 6.0 Hz, ArH), 7.28 (d, 3 H, J = 6.0 Hz, ArH and CH=), 5.13 (s, 1 H, CH), 3.99 (q, 2 H, J = 6.0 Hz, OCH_2), 1.11 (t, 3 H, J = 6.0 Hz, CH_3 in Et); ^{13}C NMR (150 MHz, DMSO-d_6) δ = 165.00, 151.80, 143.71, 137.15, 132.43, 128.86, 128.78, 102.99, 59.94, 53.81, 14.52; IR (KBr, cm^{-1}): 3302,

1698, 1675, 1384, 1183; ESI-HRMS: Calcd for $C_{13}H_{14}ClN_2O_3[M+H]^+$: 281.0693; Found: 281.0685.

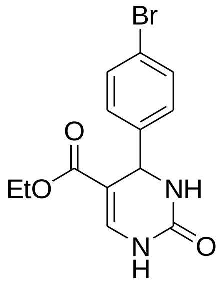

Ethyl 4-(4-bromophenyl)-2-oxo-1,2,3,4-tetrahydropyrimidine-5-carboxylate (5r). Yellow solid, m.p.: 182-184 °C; ^{1}H NMR (600 MHz, DMSO-d_6) δ = 9.24 (s, 1 H, NH), 7.71 (s, 1 H, NH), 7.53 (d, 2 H, J = 9.0 Hz, ArH), 7.27 (d, 1 H, J = 6.0 Hz, CH=), 7.21 (d, 2 H, J = 6.0 Hz, ArH), 5.11 (s, 1 H, CH), 4.00 (q, 2 H, J = 6.0 Hz, OCH_2), 1.11 (t, 3 H, J = 6.0 Hz, CH_3 in Et); ^{13}C NMR (150 MHz, DMSO-d_6) δ = 164.99, 151.78, 144.12, 137.16, 131.79, 129.14, 120.96, 102.92, 59.95, 53.88, 14.53; IR (KBr, cm^{-1}): 3297, 1698, 1674, 1380, 1190; ESI-HRMS: Calcd for $C_{13}H_{14}BrN_2O_3[M+H]^+$: 325.0188; Found: 325.0183.

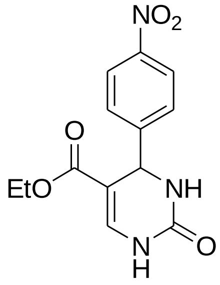

Ethyl 4-(4-nitrophenyl)-2-oxo-1,2,3,4-tetrahydropyrimidine-5-carboxylate (5s). Yellow solid, m.p.: 181-184 °C; ^{1}H NMR (600 MHz, DMSO-d_6) δ = 9.36 (d, 1 H, J = 6.0 Hz, NH), 7.87 (d, 1 H, J = 9.0 Hz, NH), 7.75-7.70 (m, 2 H, ArH), 7.56 (d, 1 H, J = 9.0 Hz, ArH), 7.49 (t, 1 H, J = 9.0 Hz, ArH), 7.29 (d, 1 H, J = 6.0 Hz, CH=), 5.87 (s, 1 H, CH=), 3.88 (q, 2 H, J = 6.0 Hz, OCH_2), 0.98 (t, 3 H, J = 6.0 Hz, CH_3 in Et); ^{13}C

NMR (150 MHz, DMSO-d_6) δ = 164.70, 151.23, 147.96, 134.51, 129.88, 129.33, 124.41, 102.28, 59.97, 49.51, 14.33; IR (KBr, cm^{-1}): 3304, 1699, 1676, 1386, 1193; ESI-HRMS: Calcd for $C_{13}H_{14}N_3O_5[M+H]^+$: 292.0933; Found: 292.0928.

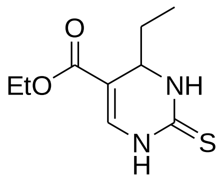

Ethyl 4-ethyl-2-thioxo-1,2,3,4-tetrahydropyrimidine-5-carboxylate (5t). White solid, m.p.: 193-195 °C; ^{1}H NMR (400 MHz, DMSO-d_6) δ = 10.14 (d, 1 H, J = 4.4 Hz, NH), 9.17 (s, 1 H, NH), 7.03 (d, 1 H, J = 6.0 Hz, CH=), 4.16-4.06 (m, 2 H, OCH_2), 1.53-1.46 (m, 2H, CH_2 in), 1.20 (t, 3 H, J = 7.2 Hz, CH_3), 0.79 (t, 3 H, J = 7.2 Hz, CH_3); ^{13}C NMR (100 MHz, DMSO-d_6) δ = 175.59, 165.11, 134.08, 103.94, 60.22, 51.77, 29.27, 14.59, 8.04; IR (KBr, cm^{-1}): 3301, 3190, 1679, 1650, 1571, 1192; ESI-HRMS: Calcd for $C_9H_{15}N_2O_2S[M+H]^+$: 215.0854; Found: 215.0848.

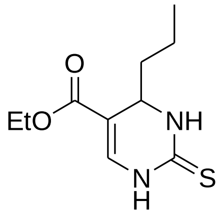

Ethyl 4-propyl-2-thioxo-1,2,3,4-tetrahydropyrimidine-5-carboxylate (5u). Pale yellow solid, m.p.: 177-180 °C; ^{1}H NMR (400 MHz, DMSO-d_6) δ = 10.15 (d, 1 H, J = 4.4 Hz, NH), 9.22 (s, 1 H, NH), 7.00 (d, 1 H, J = 5.6 Hz, CH=), 4.14-4.06 (m, 2 H, OCH_2), 1.47-1.39 (m, 2 H, CH_2), 1.30-1.18 (m, 5 H, CH_2 and CH_3), 0.86 (t, 3 H, J = 7.2 Hz, CH_3); ^{13}C NMR (100 MHz, DMSO-d_6) δ = 174.96, 164.59, 133.35, 104.21, 59.73, 50.11, 38.28, 16.30, 14.09, 13.69; IR (KBr, cm^{-1}): 3303, 3189, 1679, 1651,

1570, 1194; ESI-HRMS: Calcd for $C_{10}H_{17}N_2O_2S[M+H]^+$: 229.1011; Found: 229.1005.

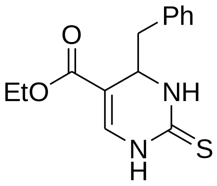

Ethyl 4-benzyl-2-thioxo-1,2,3,4-tetrahydropyrimidine-5-carboxylate (5v). Pale yellow solid, m.p.:171-172 ^oC; ^1H NMR (400 MHz, DMSO-d$_6$) δ = 9.82 (d, 1 H, *J* = 4.4 Hz, NH), 9.20 (s, 1 H, NH), 7.27-7.21 (m, 3 H, ArH), 7.04 (d, 2 H, *J* = 6.8 Hz, ArH), 6.79 (d, 1 H, *J* = 5.6 Hz, CH=), 4.44 (d, 1 H, *J* = 3.2 Hz, CH), 4.13-4.06 (m, 2 H, OCH_2), 2.78 (d, 2 H, *J* = 4.0 Hz, $PhCH_2$), 1.19 (t, 3 H, *J* = 7.2 Hz, CH_3 in Et); ^{13}C NMR (100 MHz, DMSO-d$_6$) δ = 175.54, 165.10, 136.52, 134.05, 130.41, 128.34, 126.87, 103.79, 60.18, 52.32, 40.01, 14.61; IR (KBr, cm^{-1}): 3305, 3188, 1667, 1570, 1175; ESI-HRMS: Calcd for $C_{14}H_{17}N_2O_2S[M+H]^+$: 277.1011; Found: 277.1018.

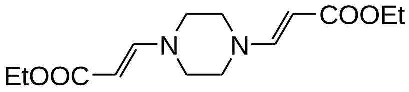

(2*E*,2'*E*)-diethyl 3,3'-(piperazine-1,4-diyl)diacrylate (6a). 80mg, 95 %; White solid, m.p.: 206-207 ^oC; ^1H NMR (400 MHz, $CDCl_3$) δ = 7.31 (d, 2 H, *J* = 13.2 Hz, CH=×2), 4.65 (d, 2 H, *J* = 13.2 Hz, CH=×2), 4.07 (q, 4 H, *J* = 6.8 Hz OCH_2×2), 3.20 (s, 8 H, CH_2CH_2×2), 1.19 (t, 6 H, *J* = 6.8 Hz, CH_3×2); ^{13}C NMR (100 MHz, $CDCl_3$) δ = 169.17, 151.02, 87.11, 59.17, 47.47, 14.51; IR (KBr, cm^{-1}): 2977, 2916, 1685, 1378; ESI-HRMS: Calcd for $C_{14}H_{23}N_20_4[M+H]^+$: 283.1658; Found: 283.1650.

^{1}H and ^{13}C NMR of **5a**

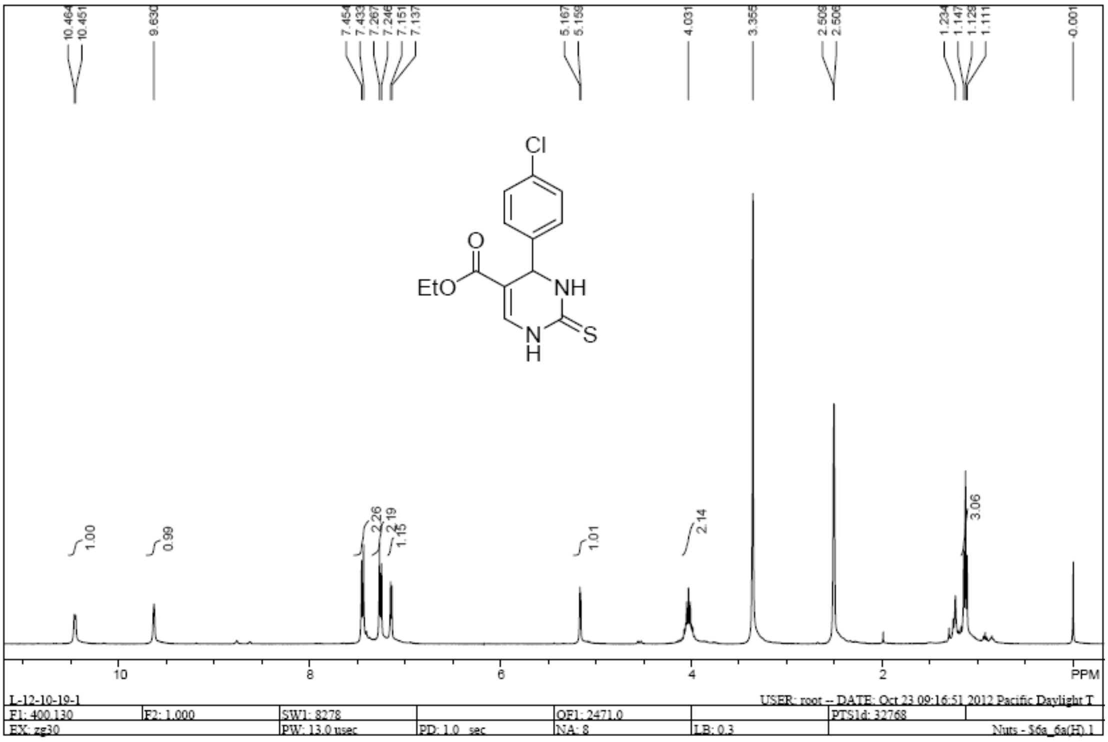

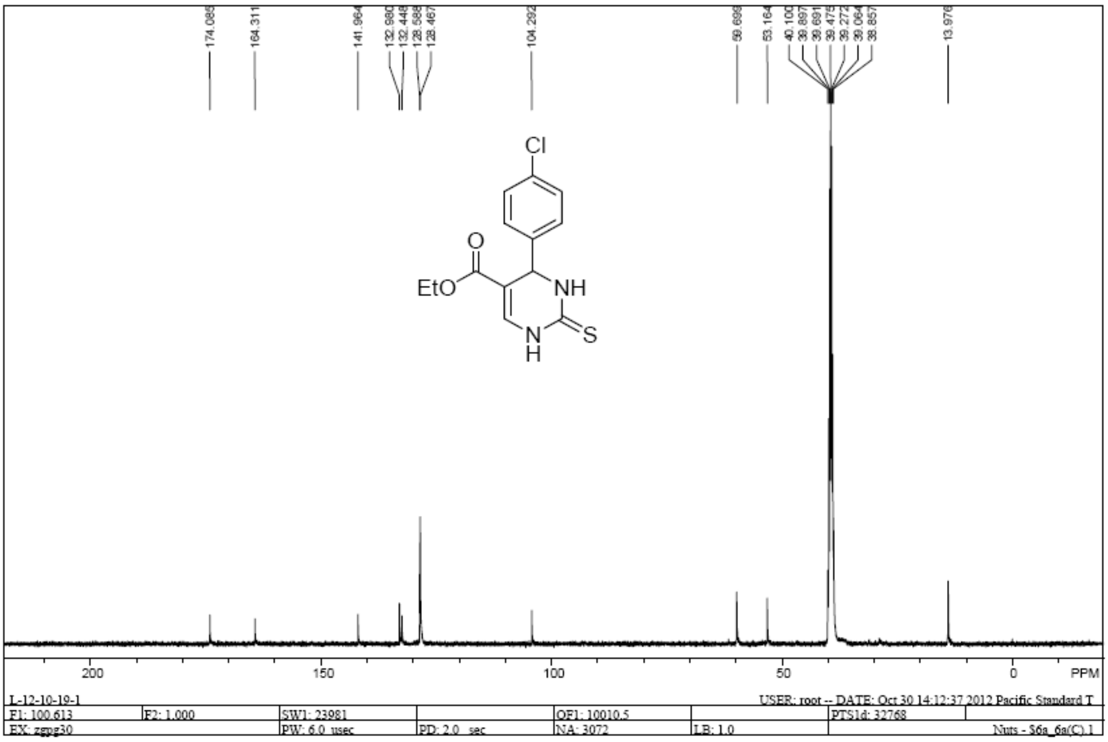

^{1}H and ^{13}C NMR of **5b**

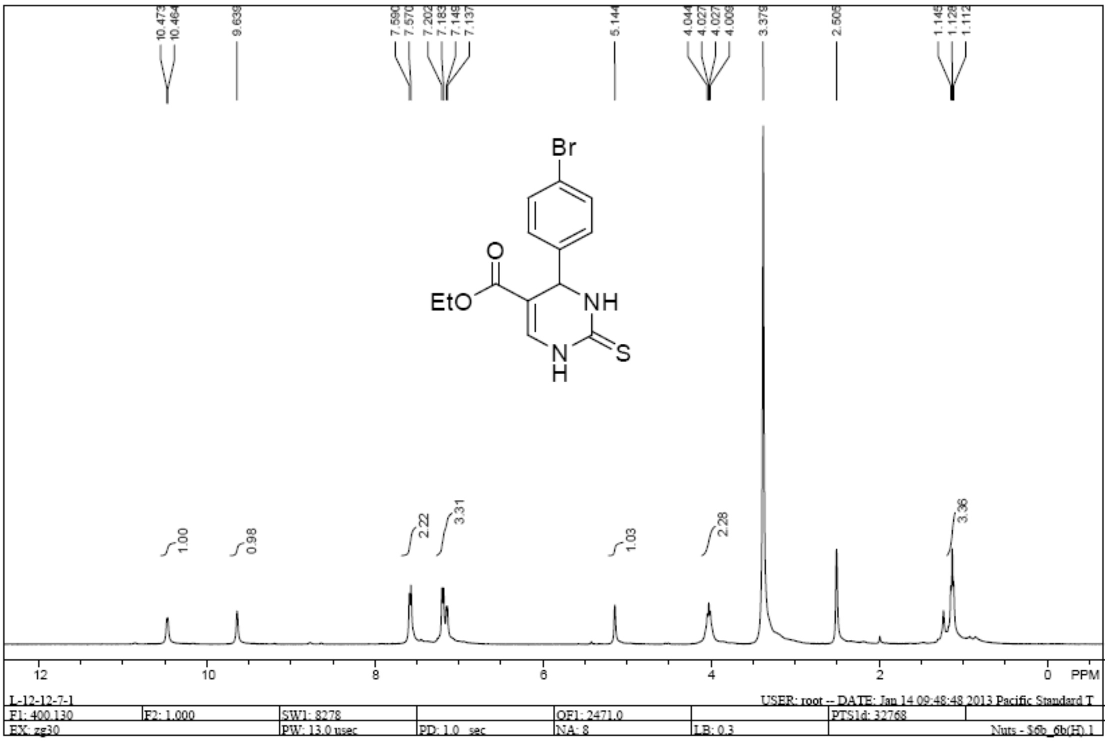

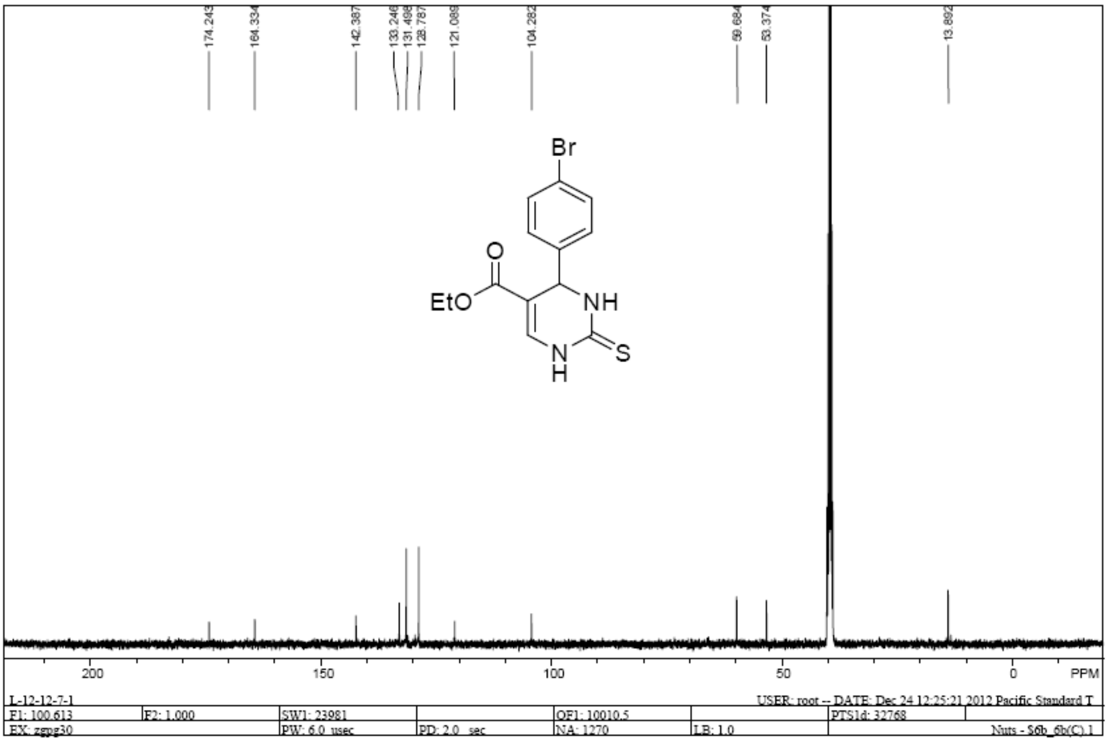

^{1}H and ^{13}C NMR of **5c**

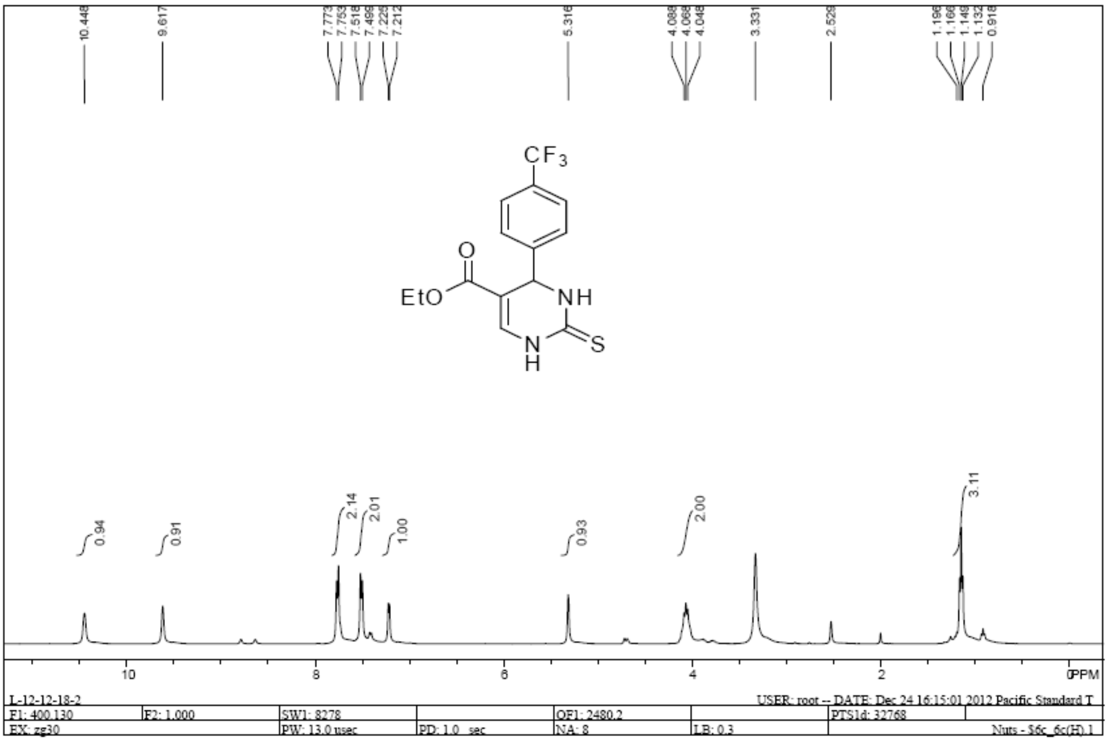

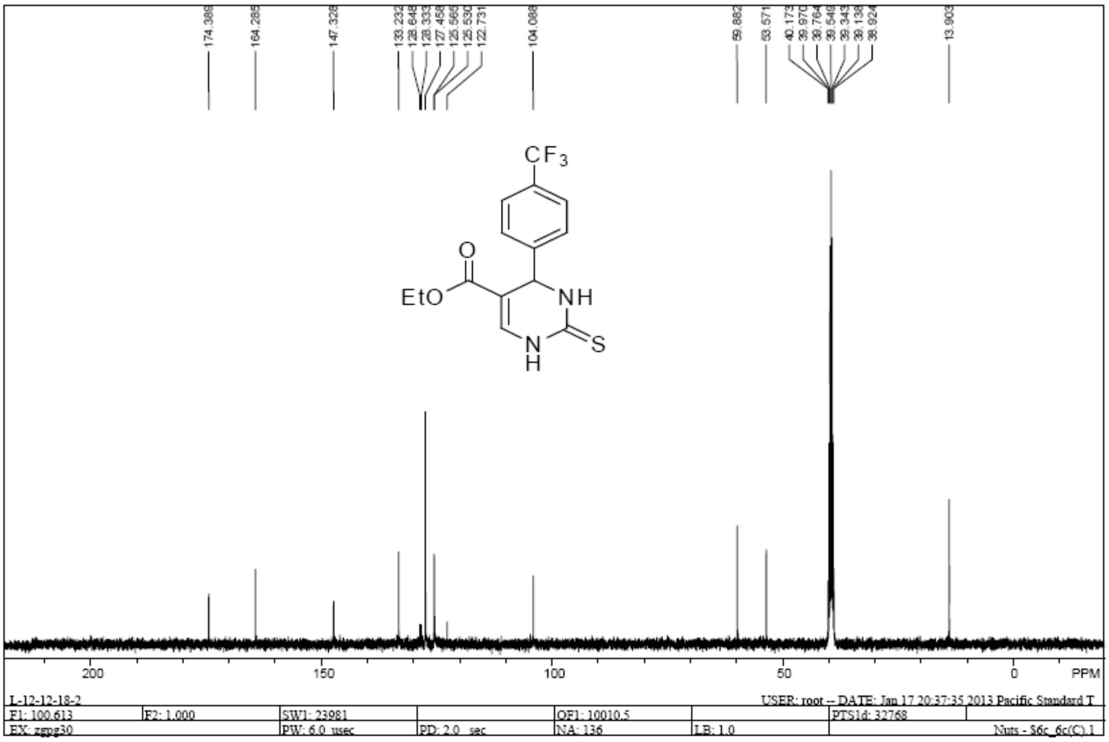

^{1}H and ^{13}C NMR of **5d**

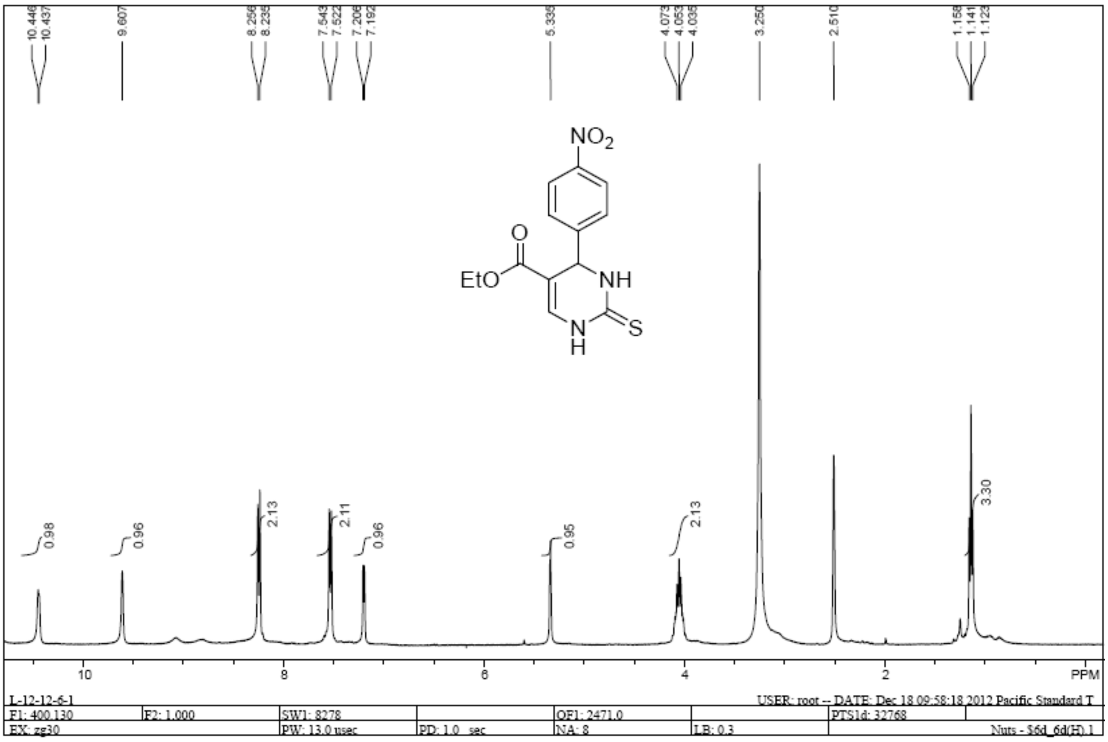

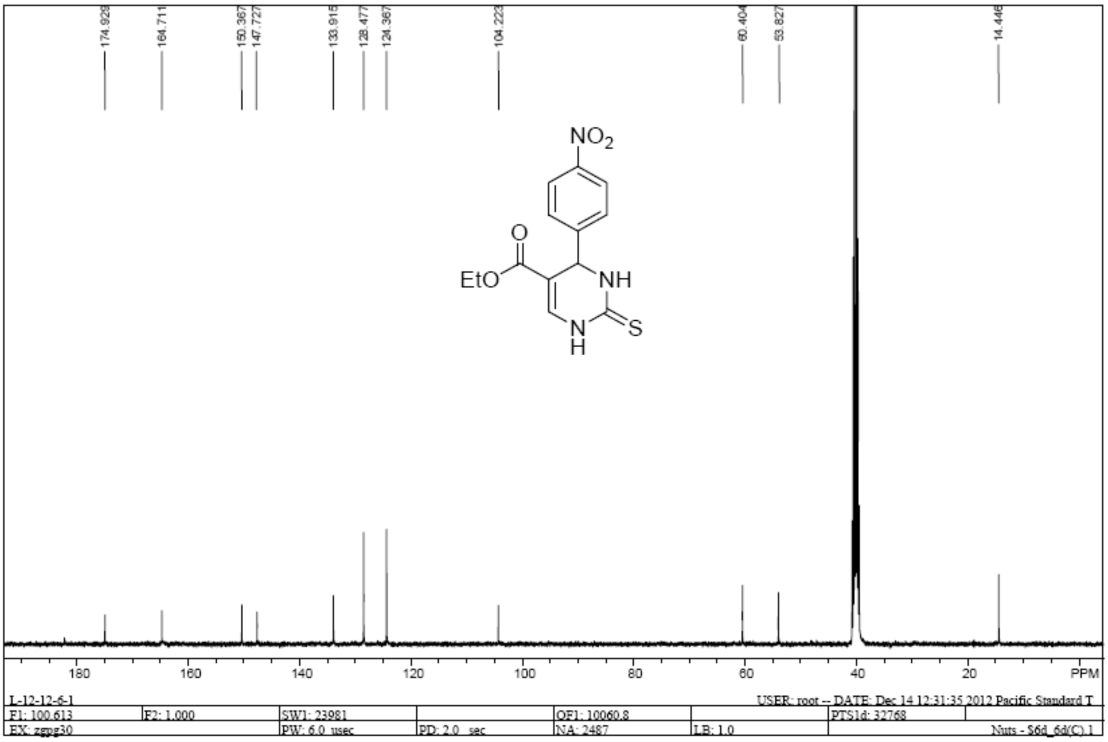

^{1}H and ^{13}C NMR of **5e**

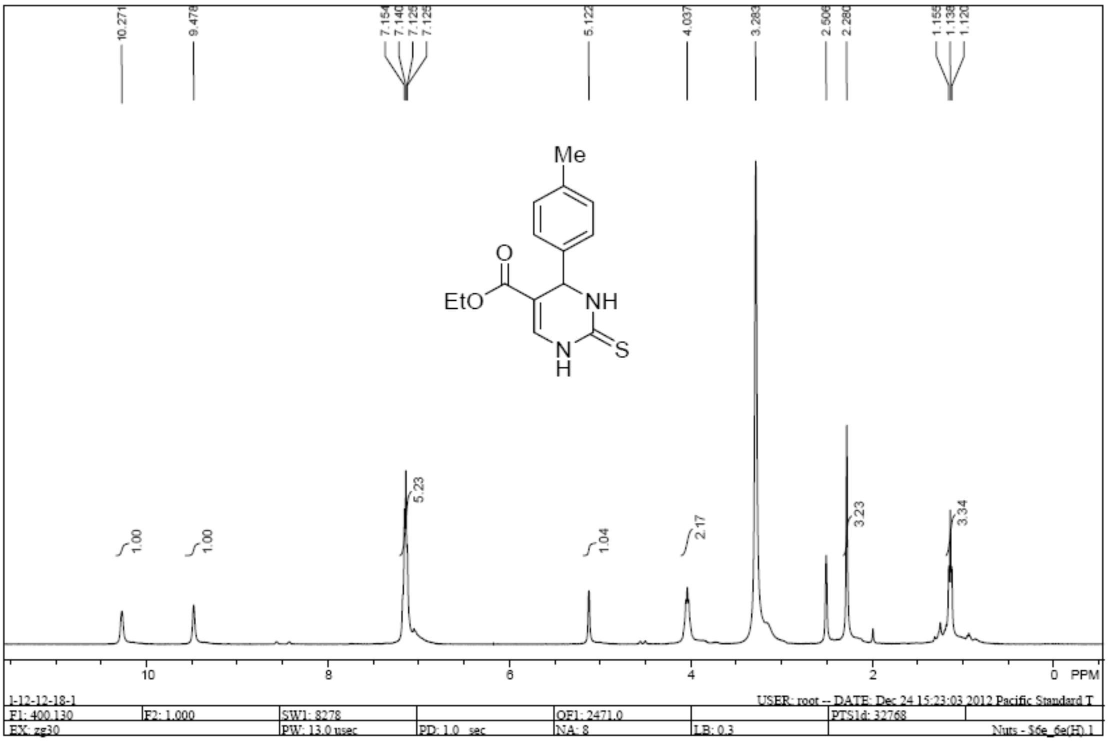

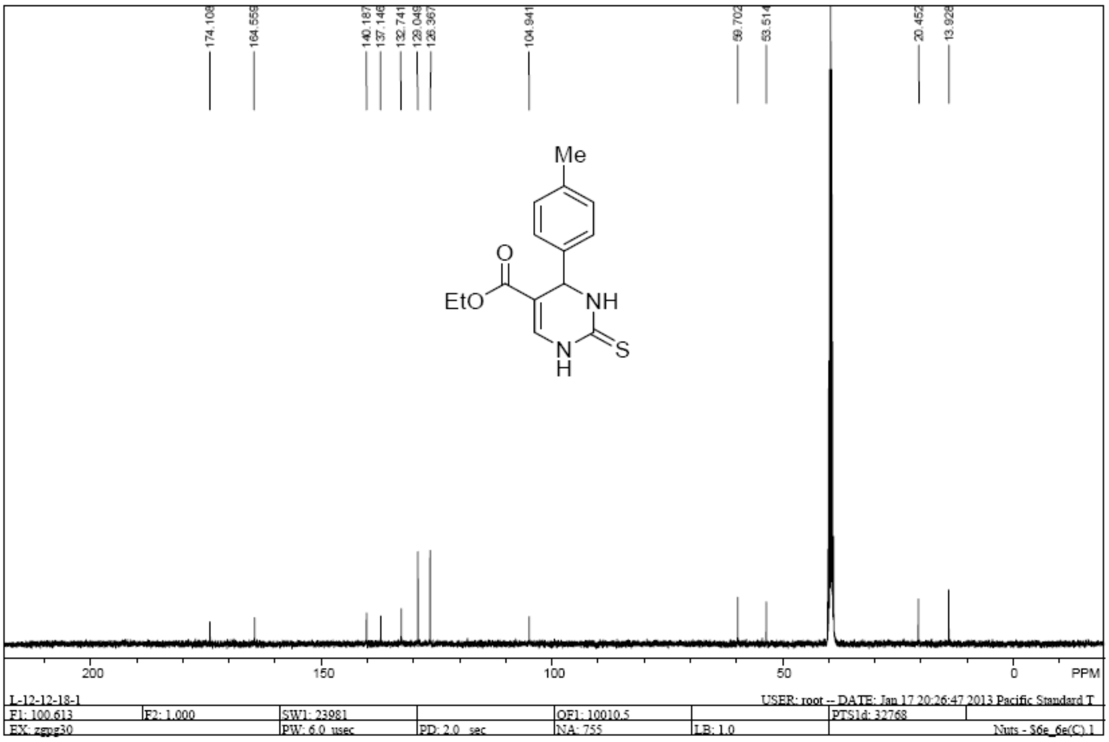

^{1}H and ^{13}C NMR of **5f**

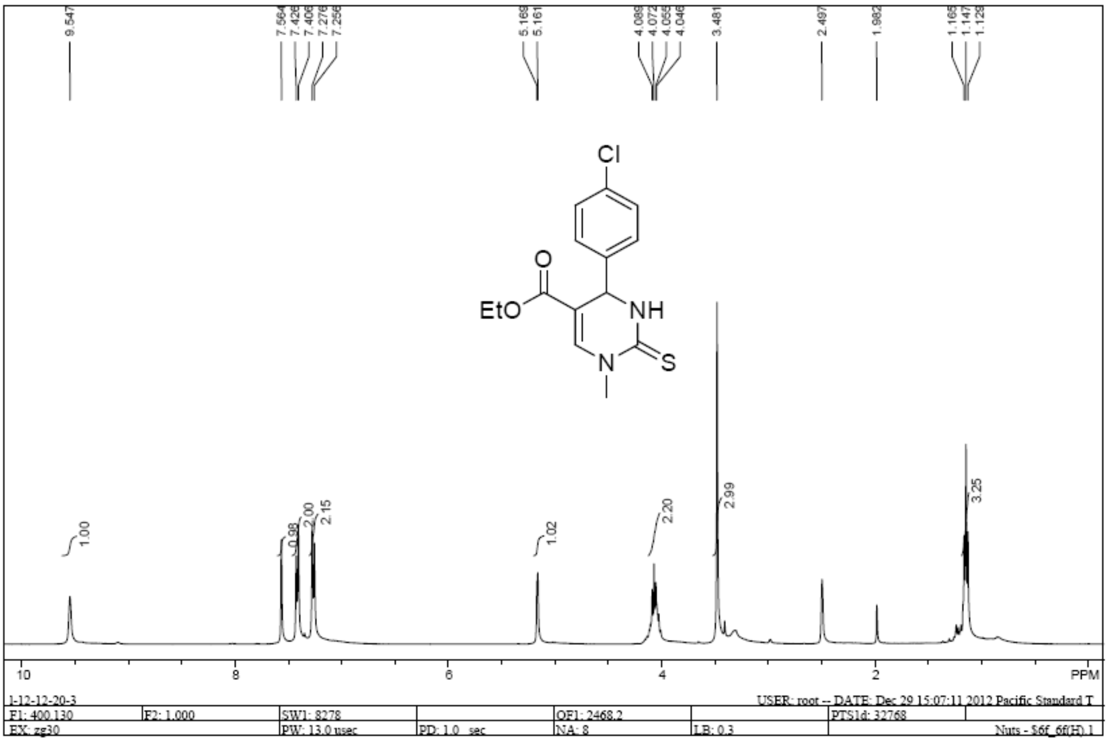

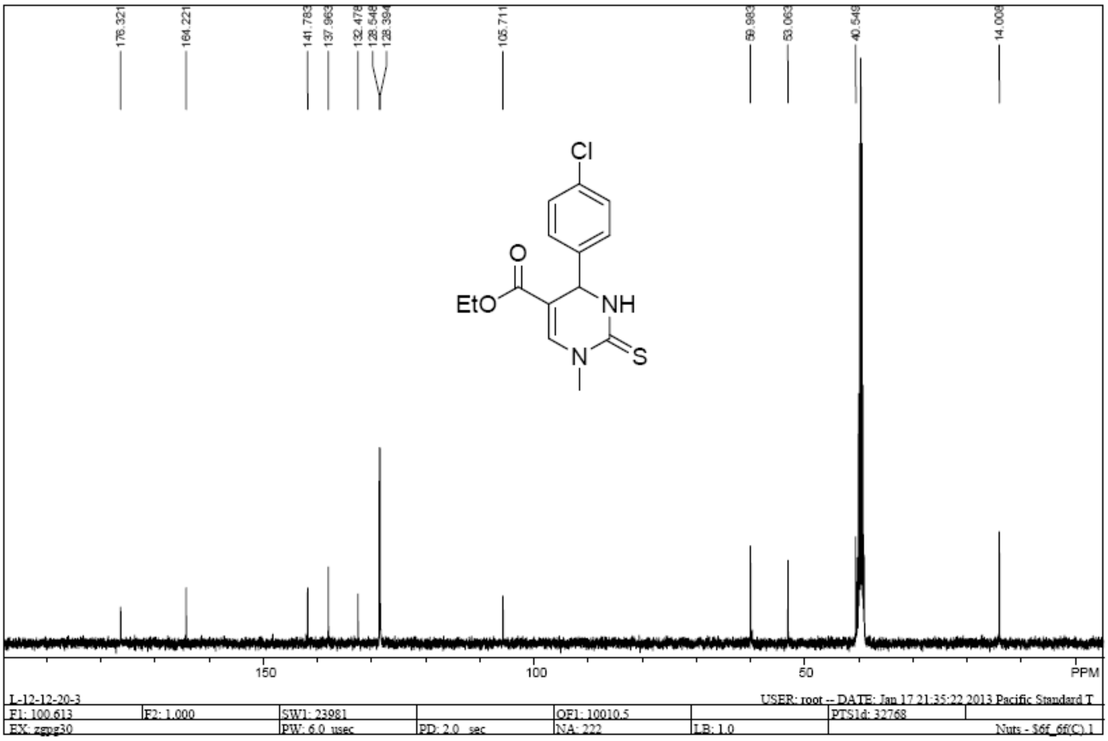

^{1}H and ^{13}C NMR of **5g**

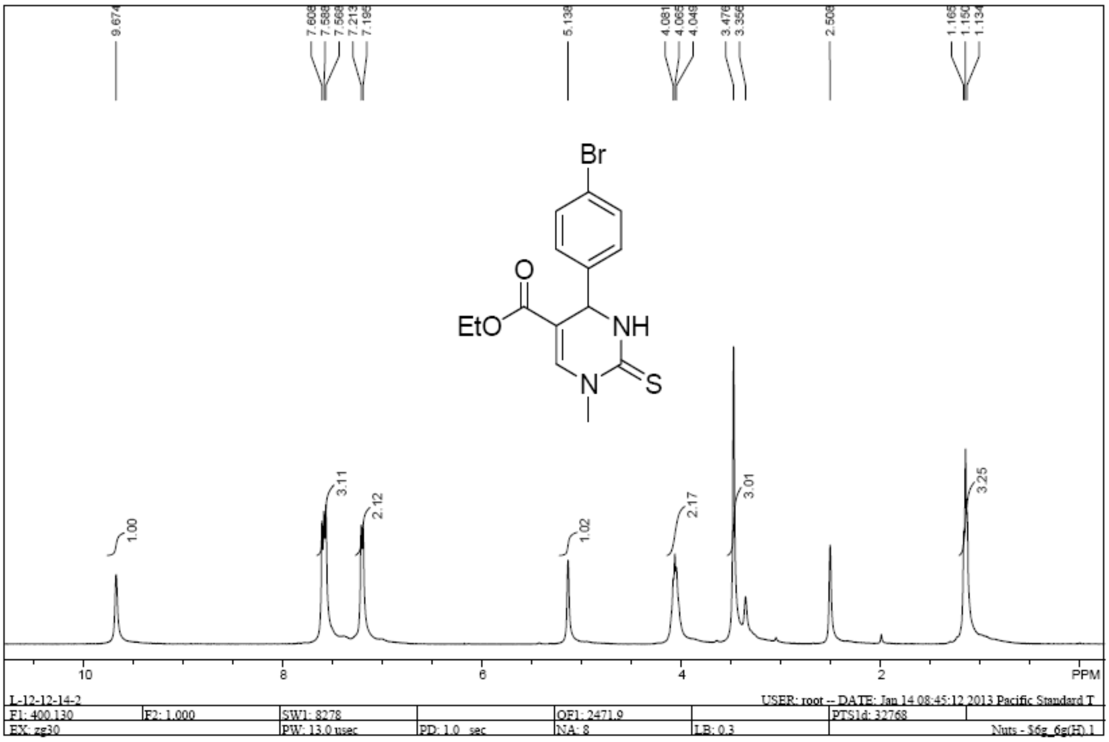

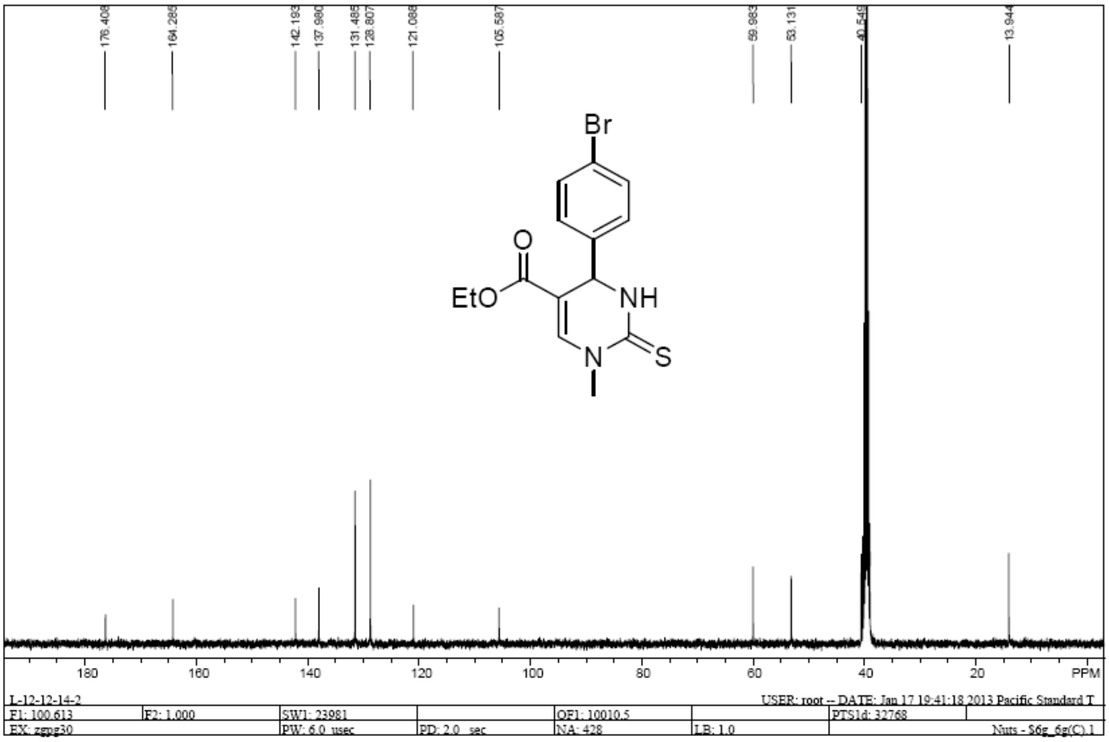

^{1}H and ^{13}C NMR of **5h**

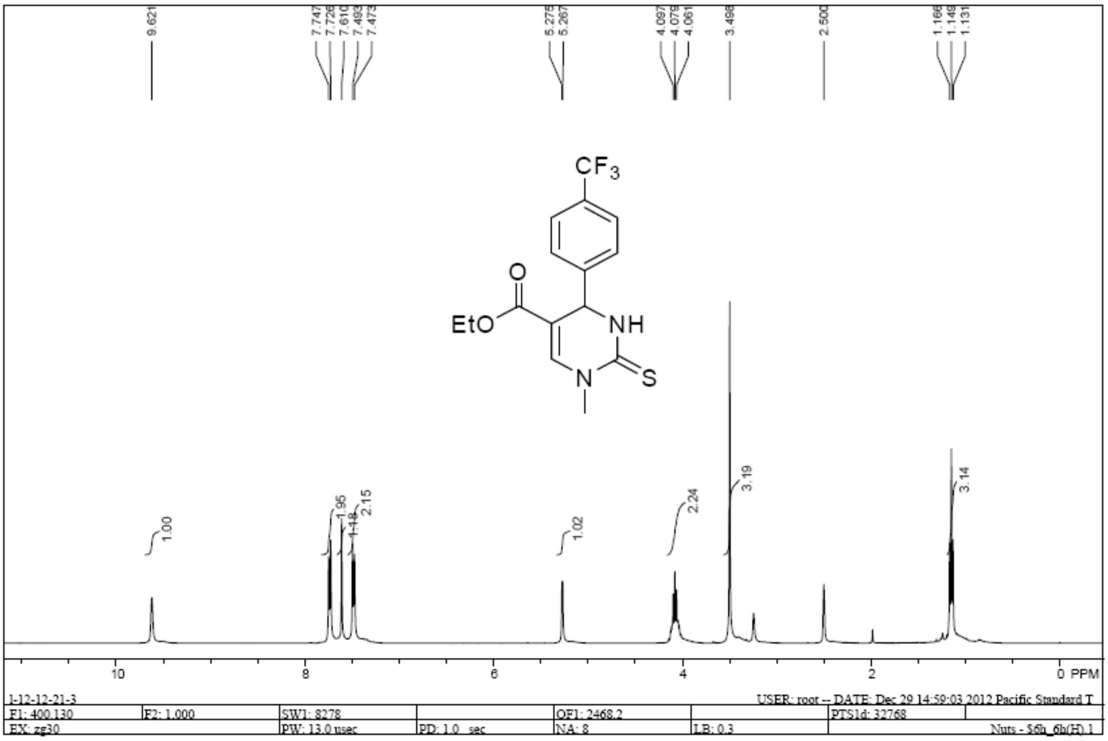

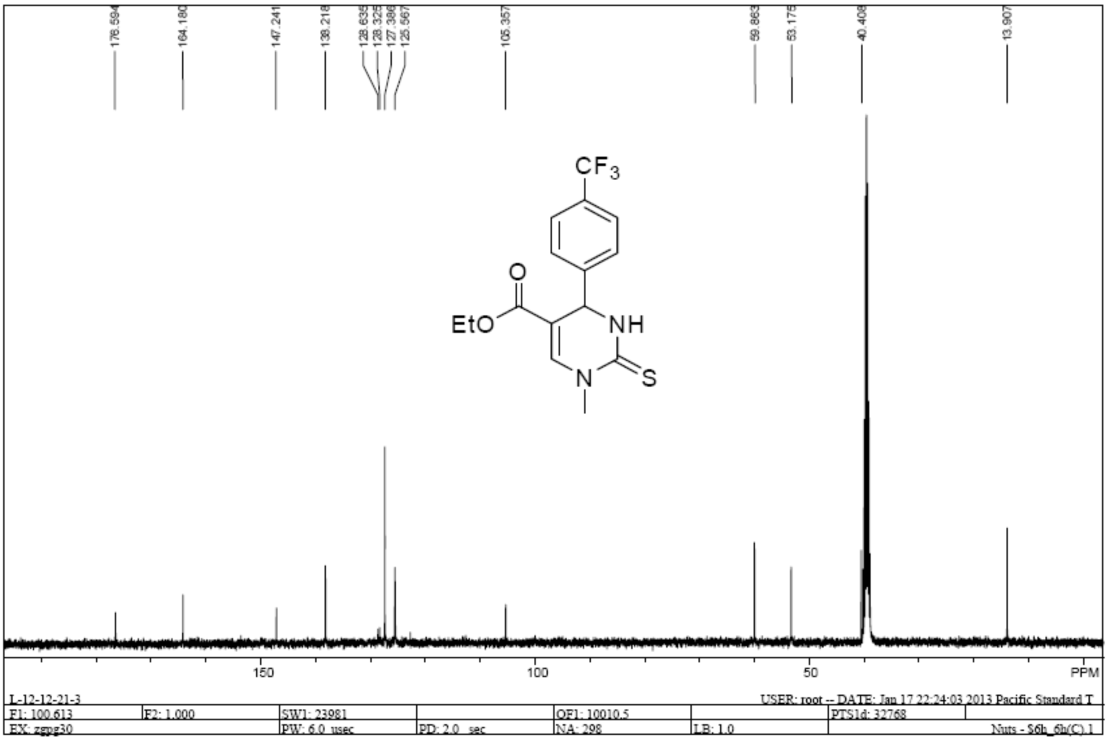

^{1}H and ^{13}C NMR of **5i**

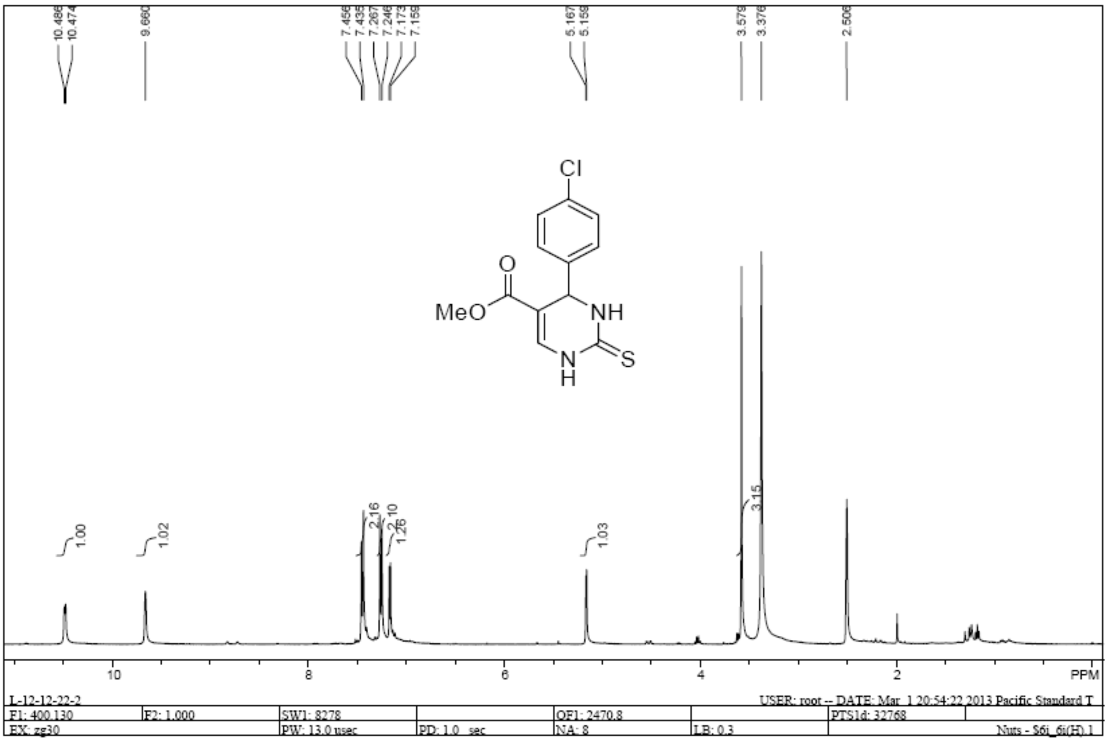

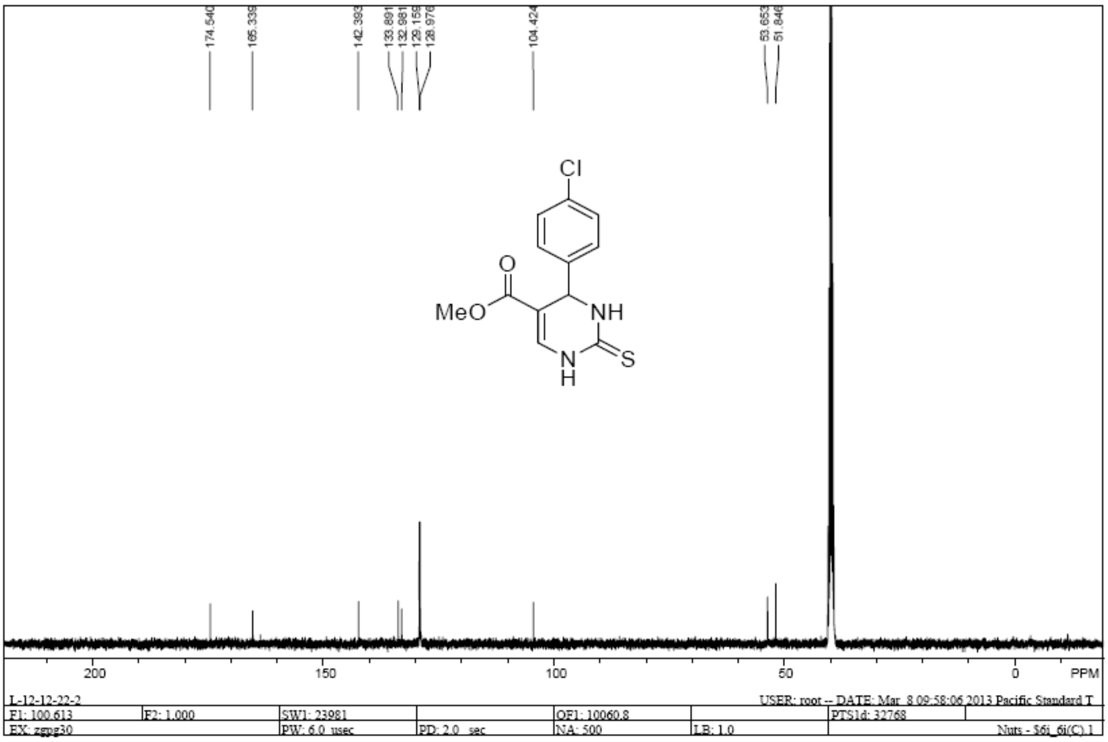

^{1}H and ^{13}C NMR of **5j**

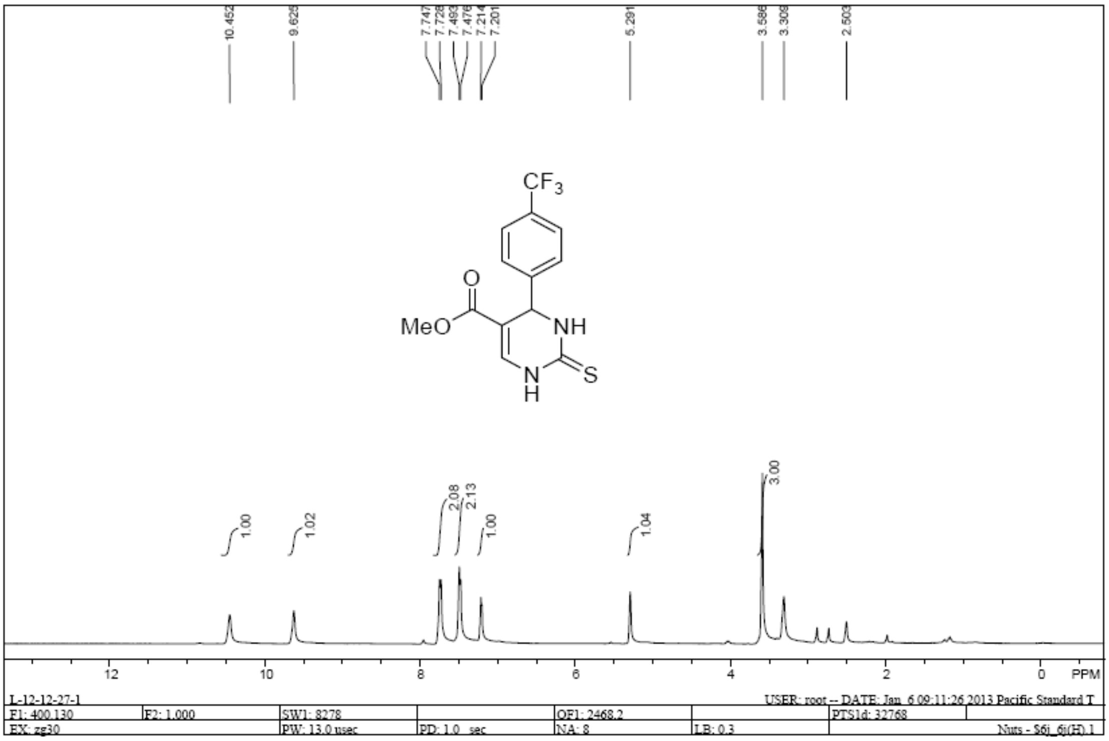

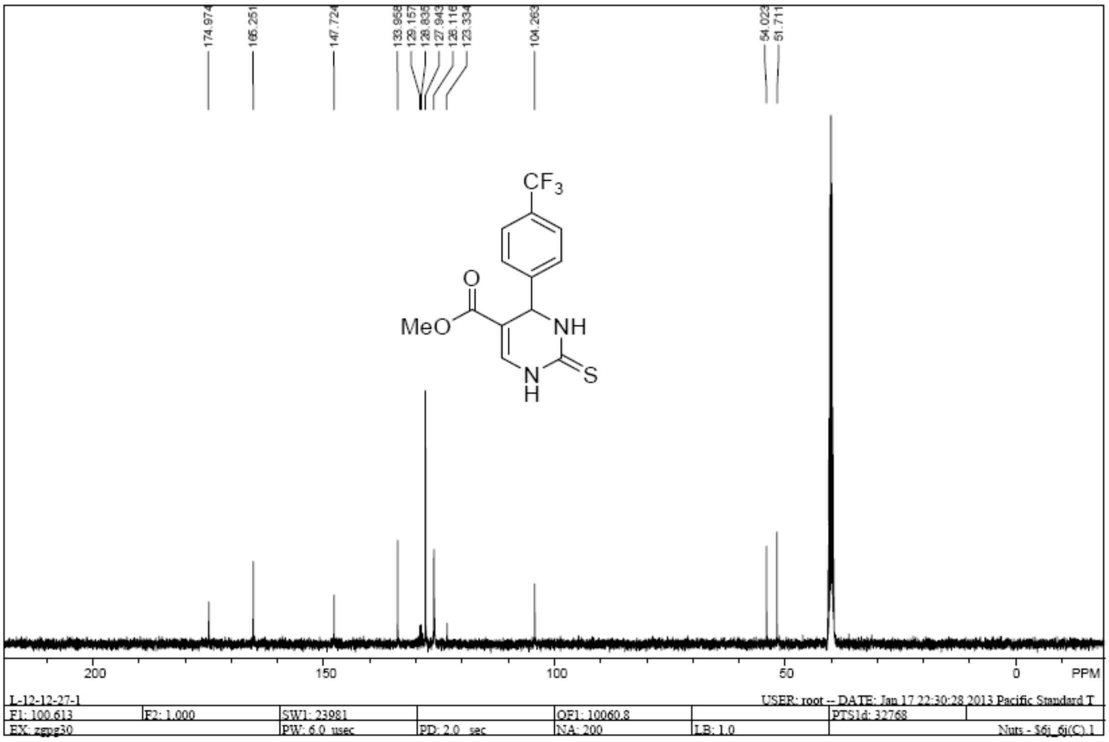

^{1}H and ^{13}C NMR of **5k**

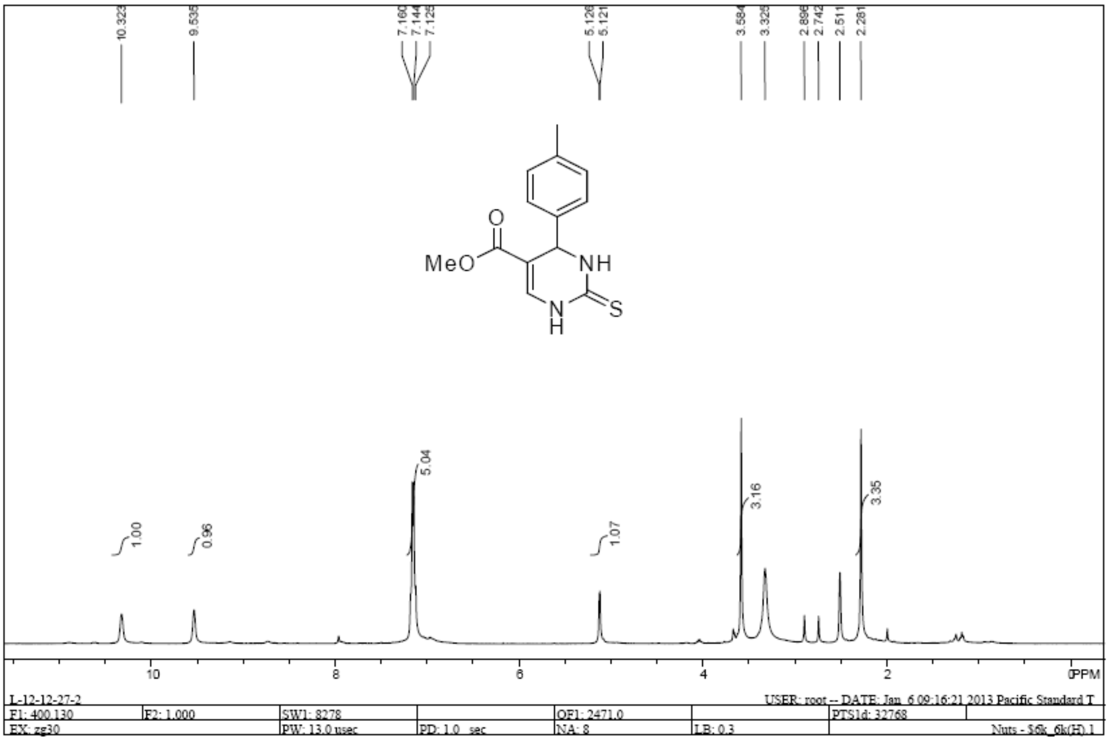

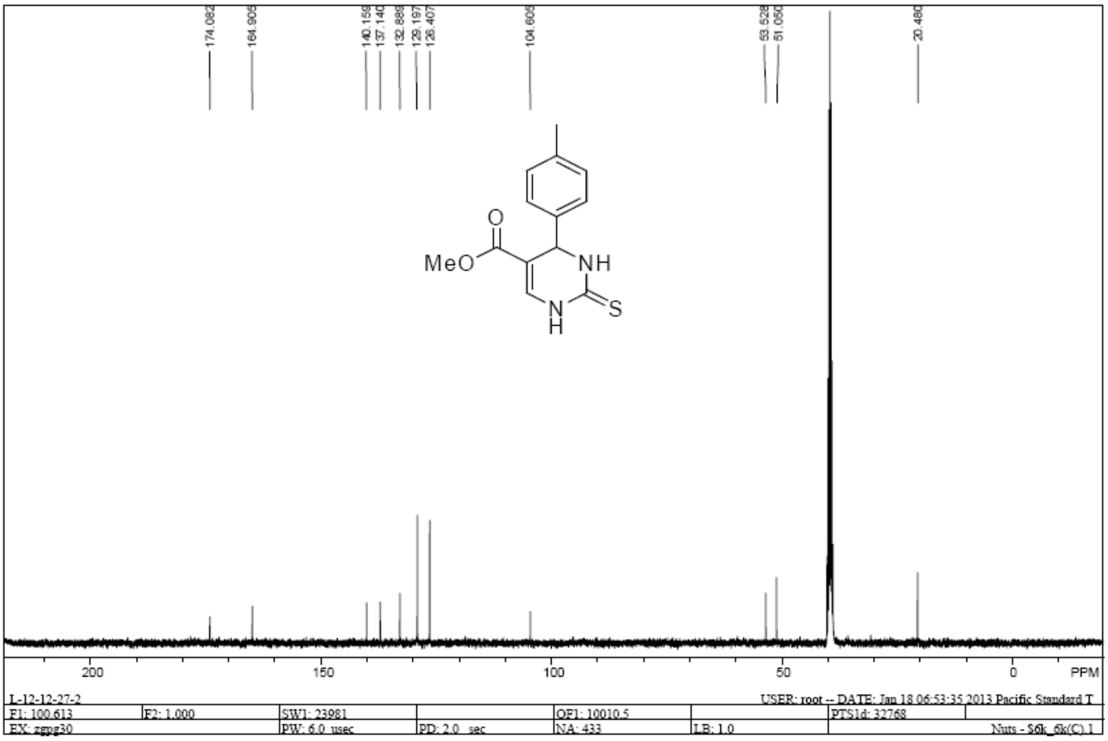

^{1}H and ^{13}C NMR of **5l**

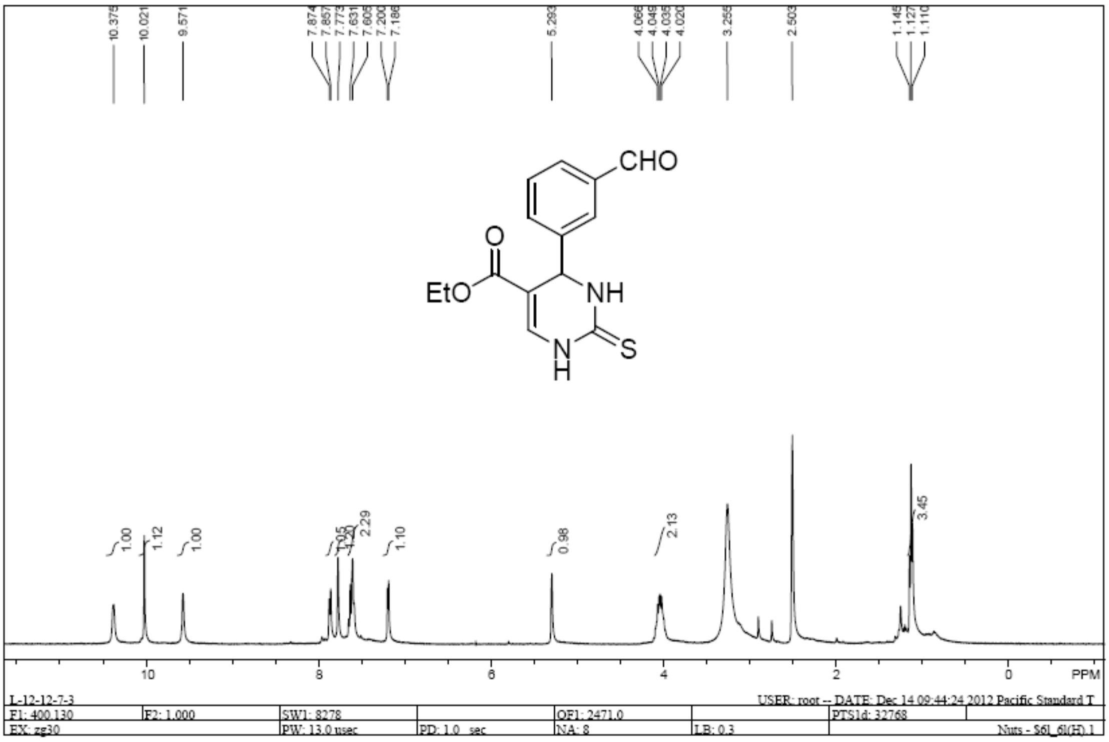

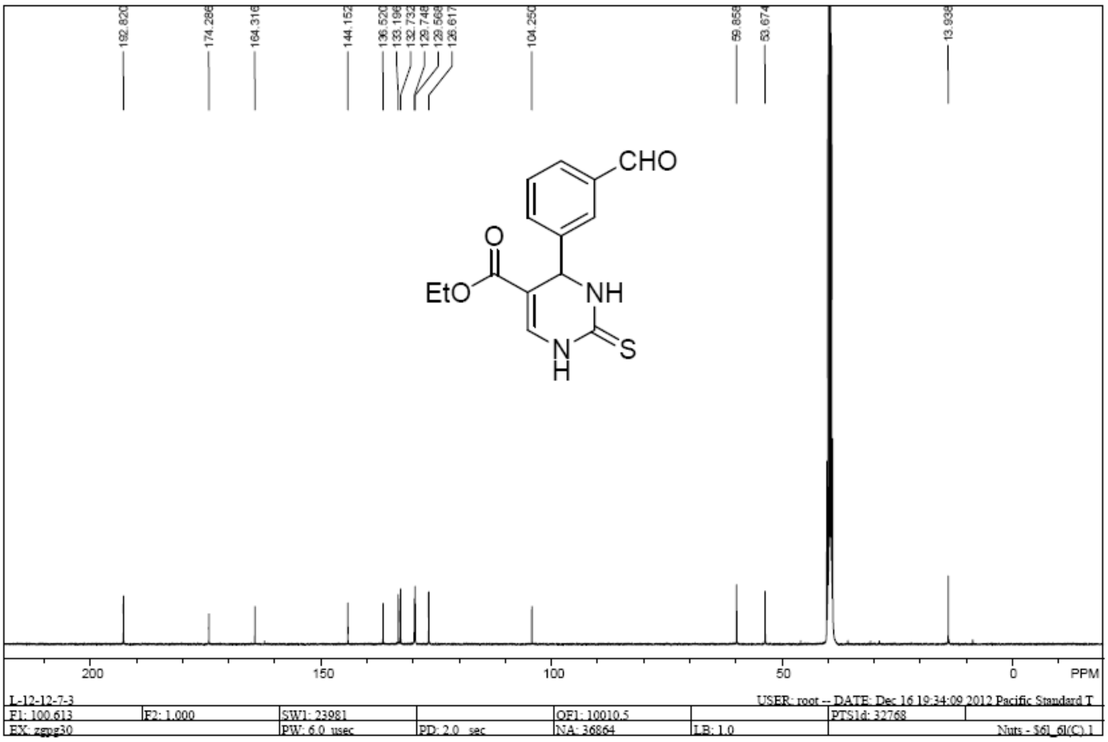

^1H and ^{13}C NMR of **5m**

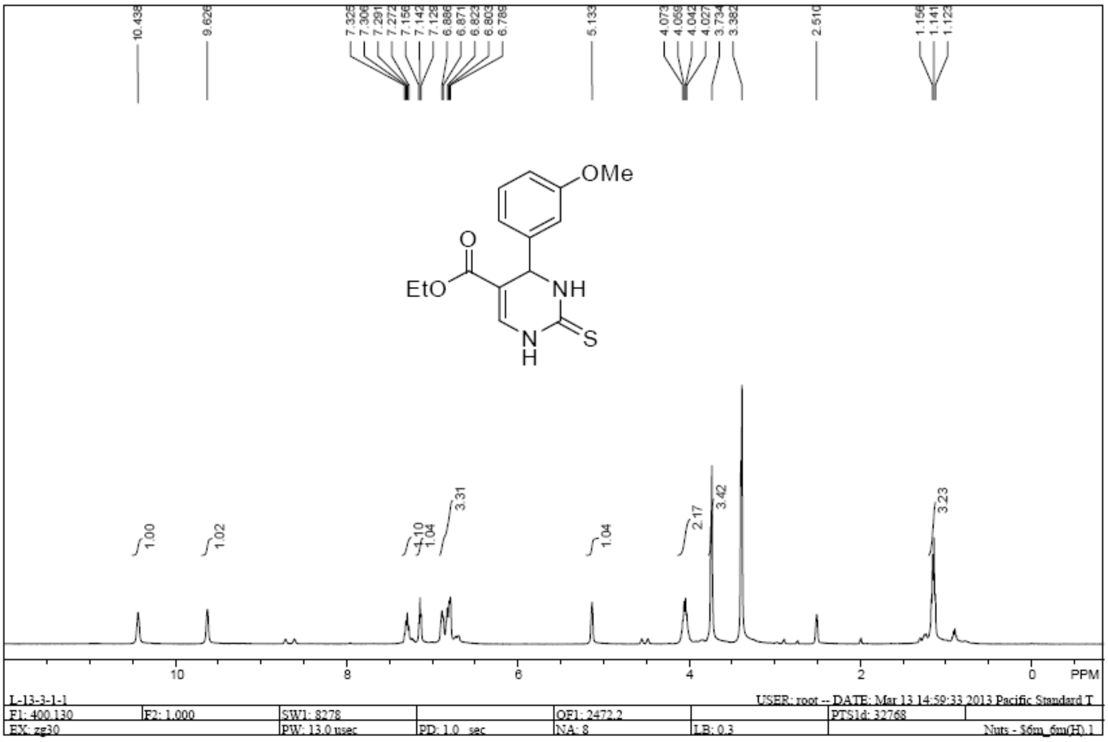

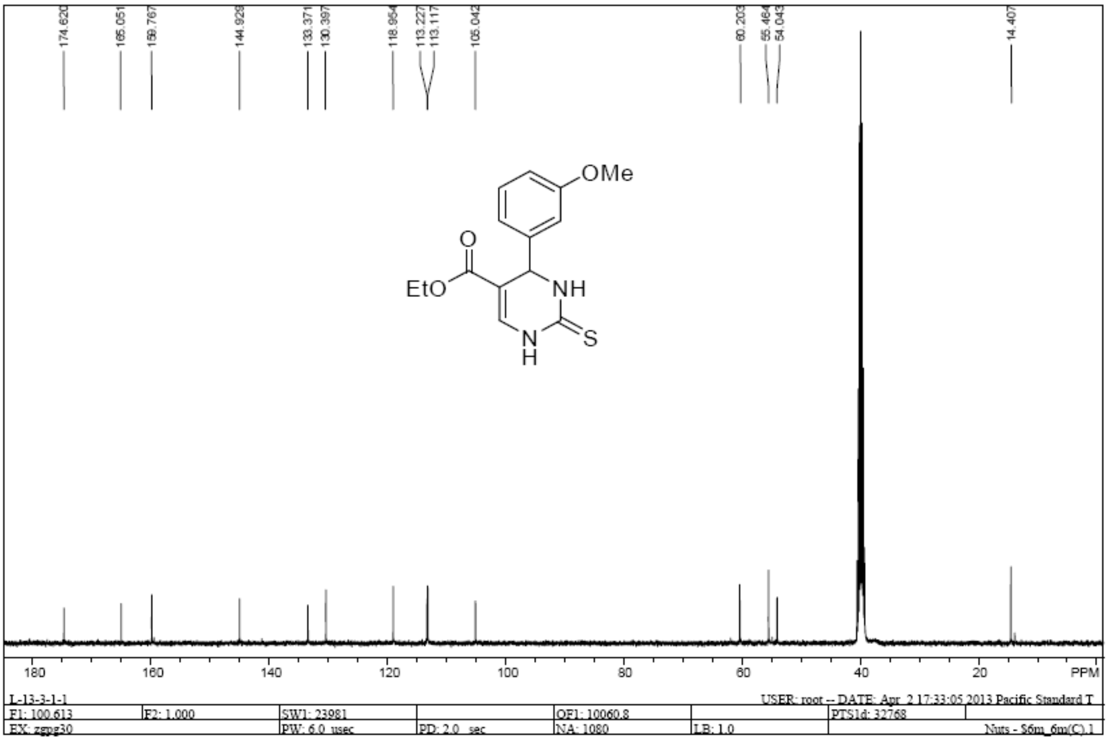

^{1}H and ^{13}C NMR of **5n**

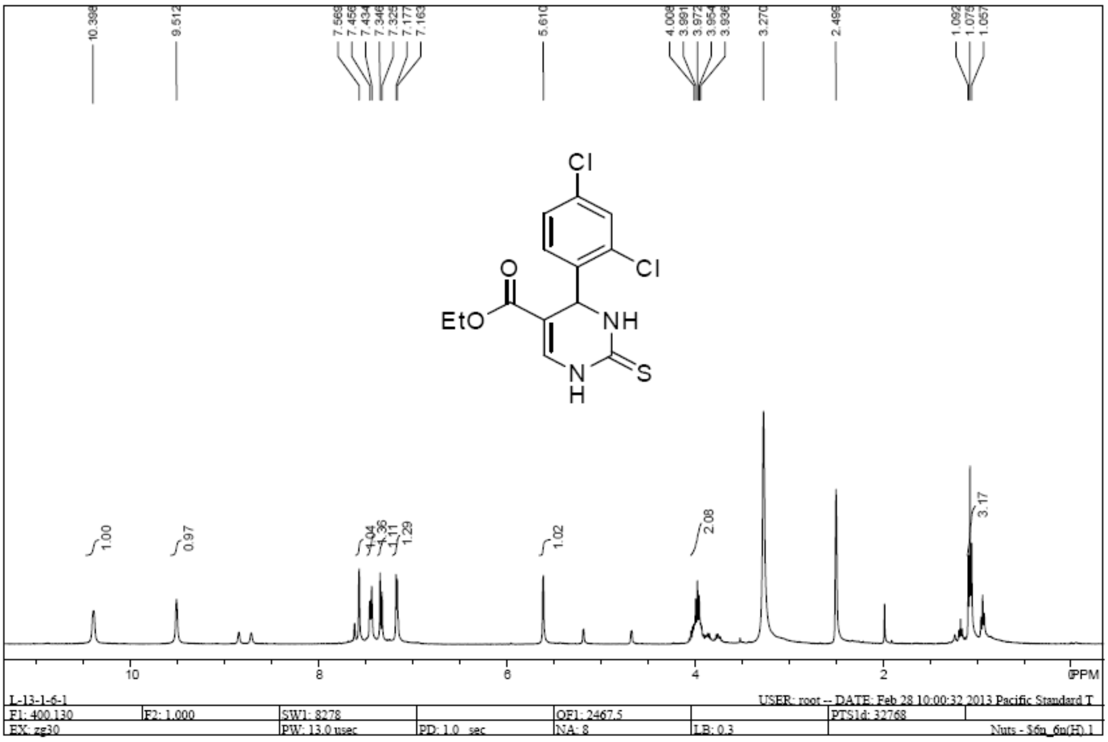

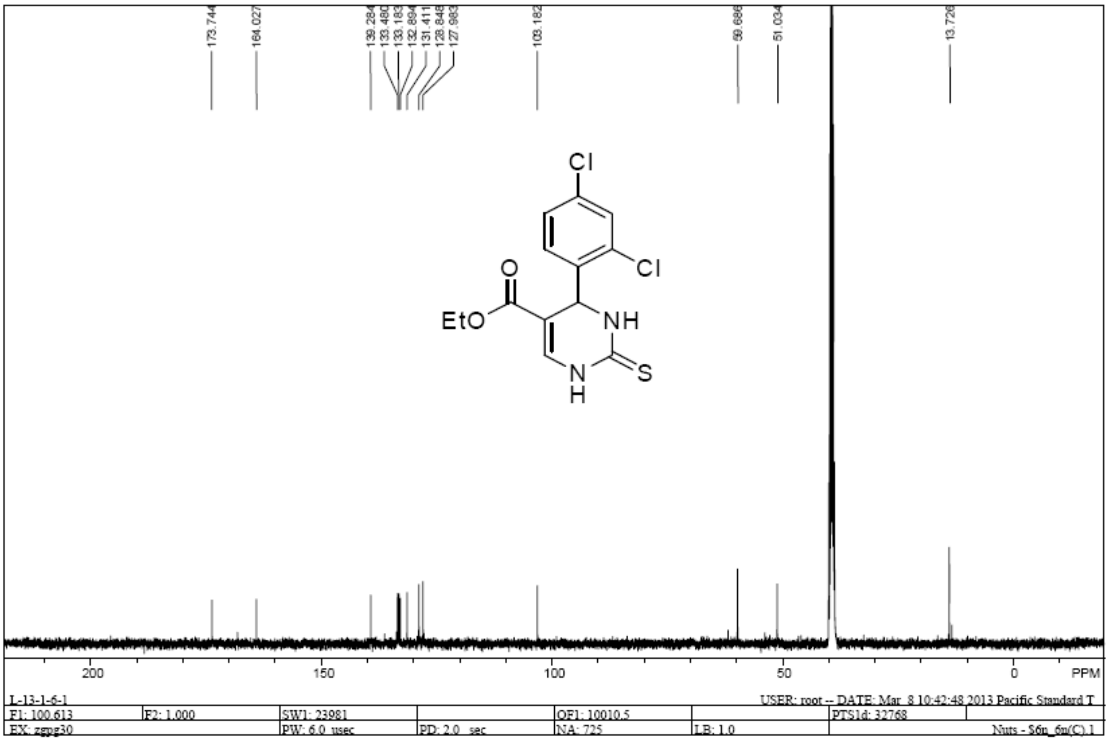

^{1}H and ^{13}C NMR of **5o**

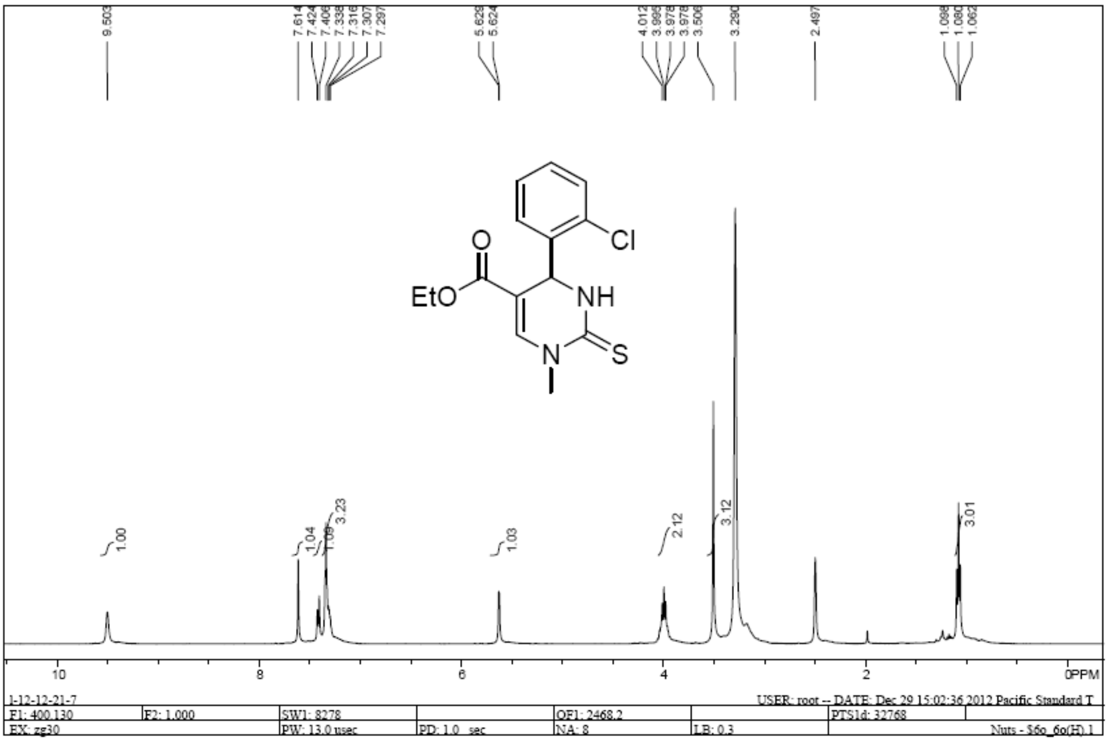

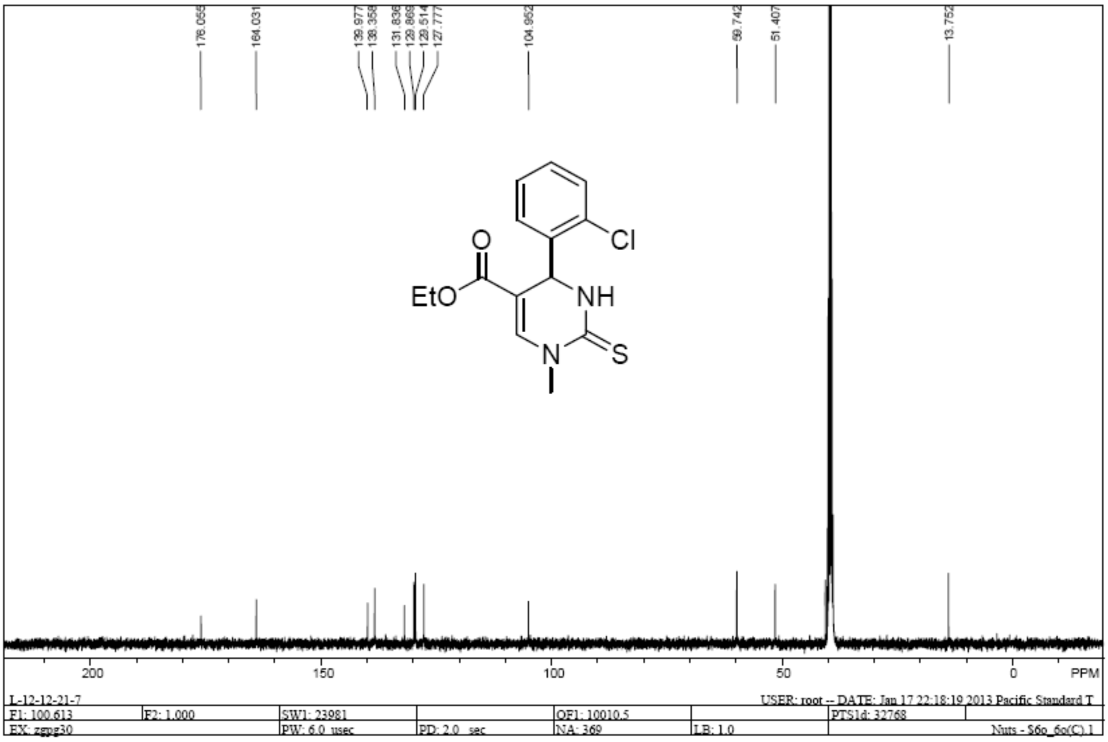

^{1}H and ^{13}C NMR of **5p**

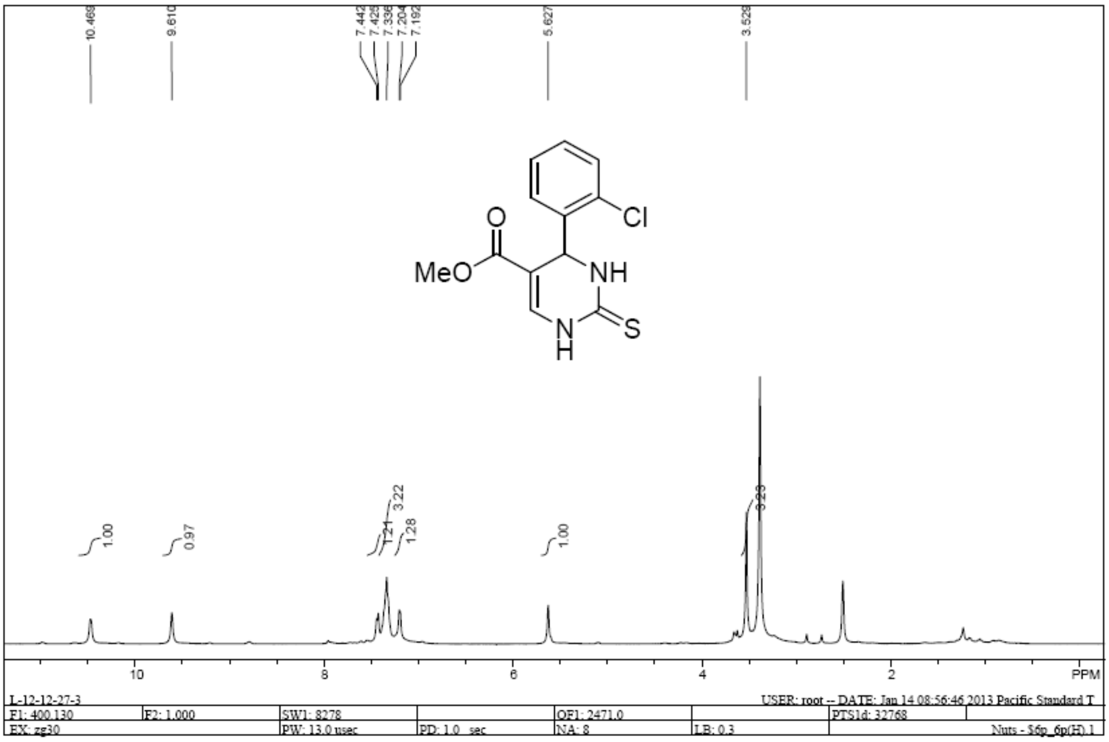

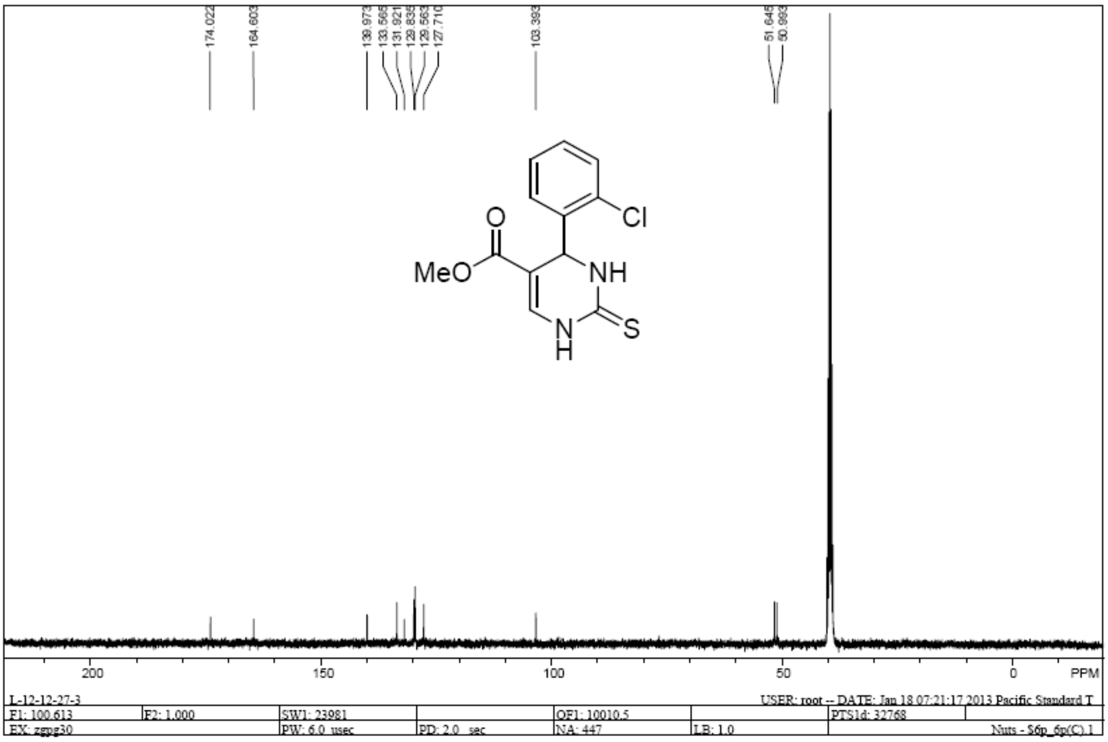

^{1}H and ^{13}C NMR of **5q** (**5q-5s** were recorded in 600 MHz NMR)

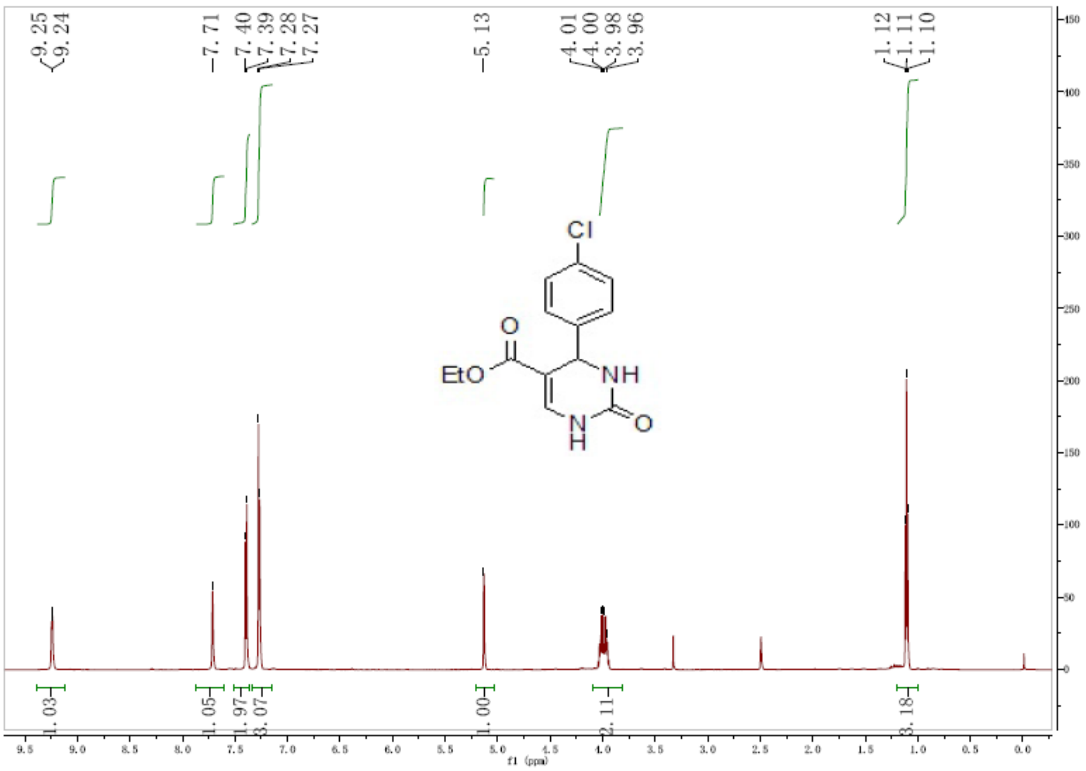

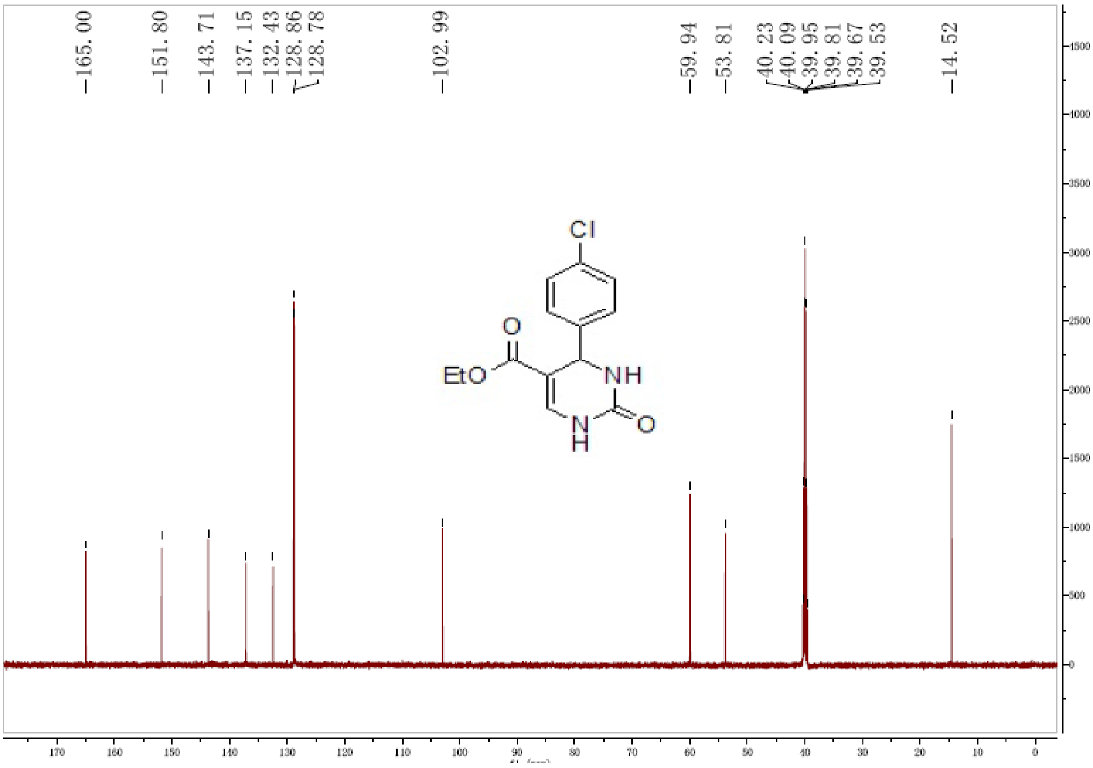

^{1}H and ^{13}C NMR of **5r**

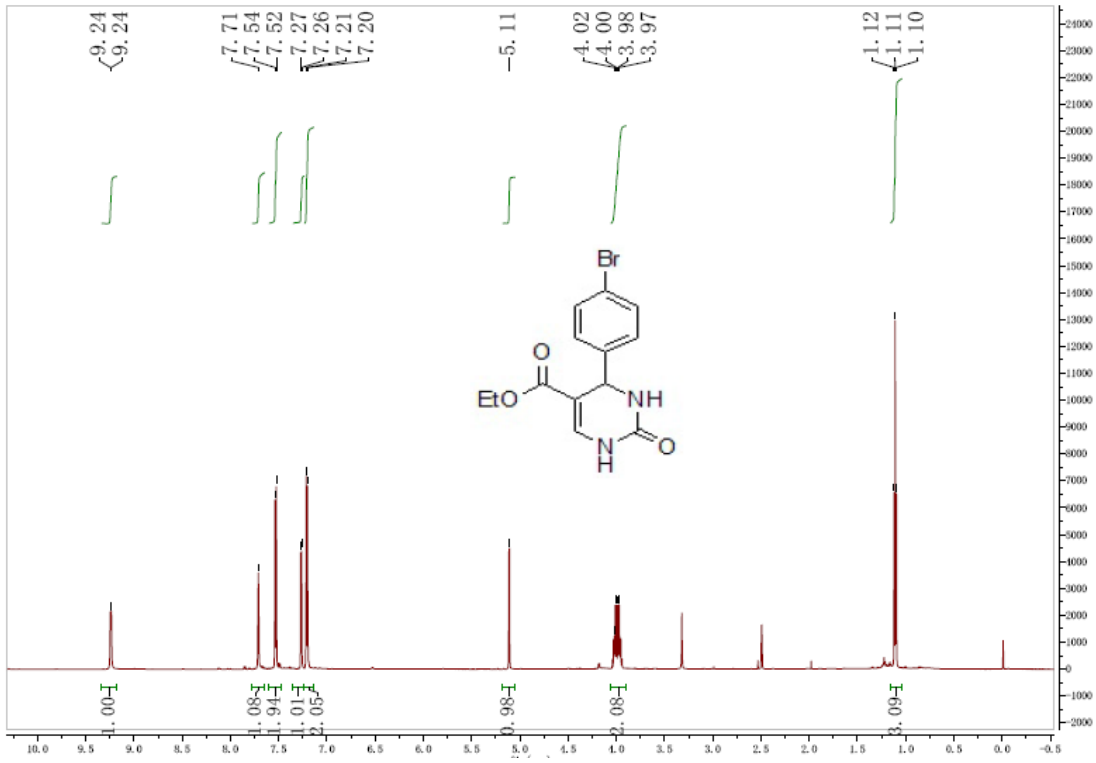

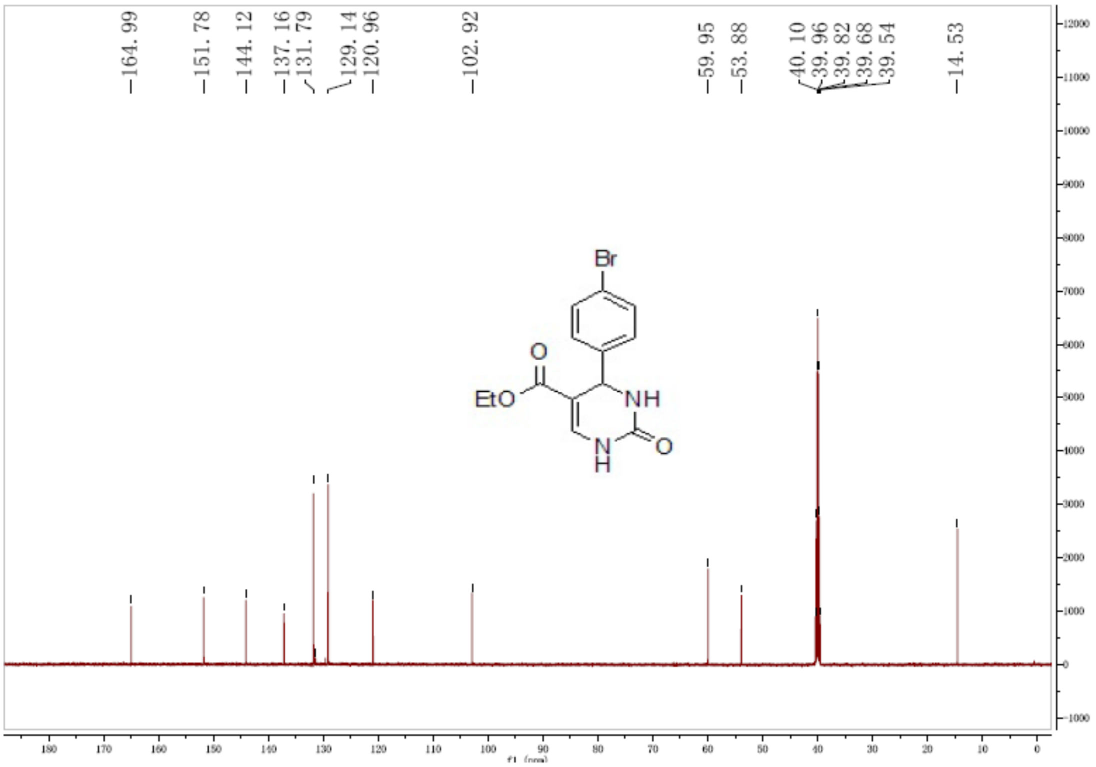

^{1}H and ^{13}C NMR of **5s**

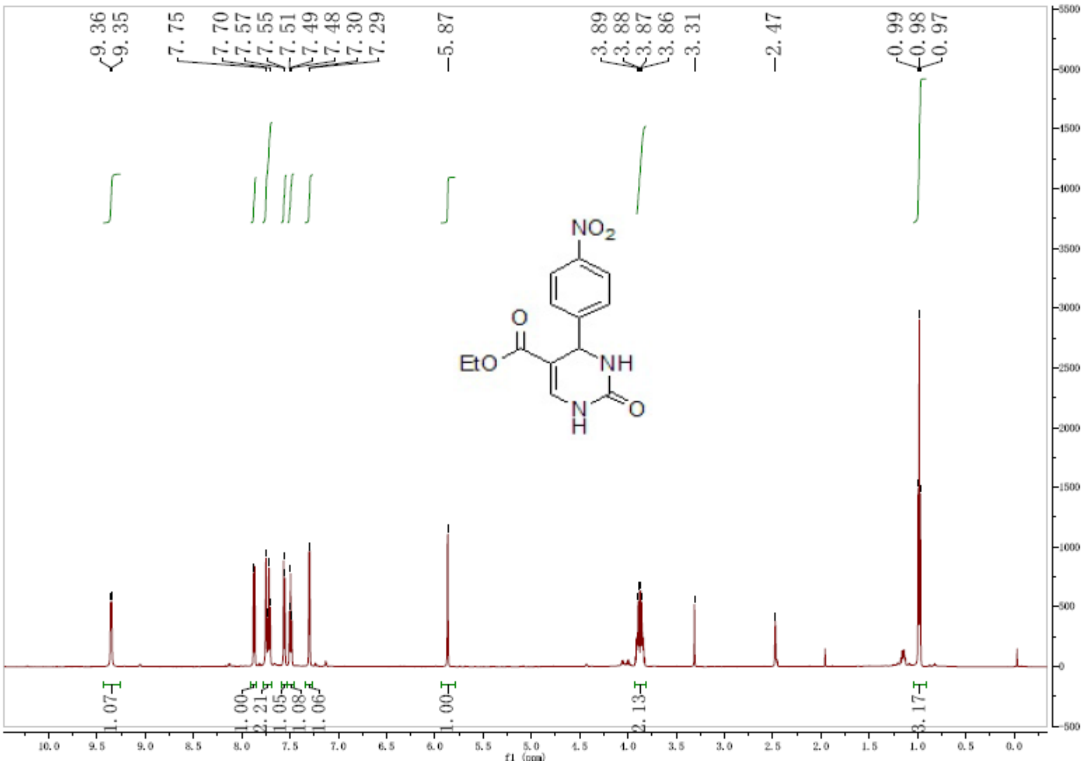

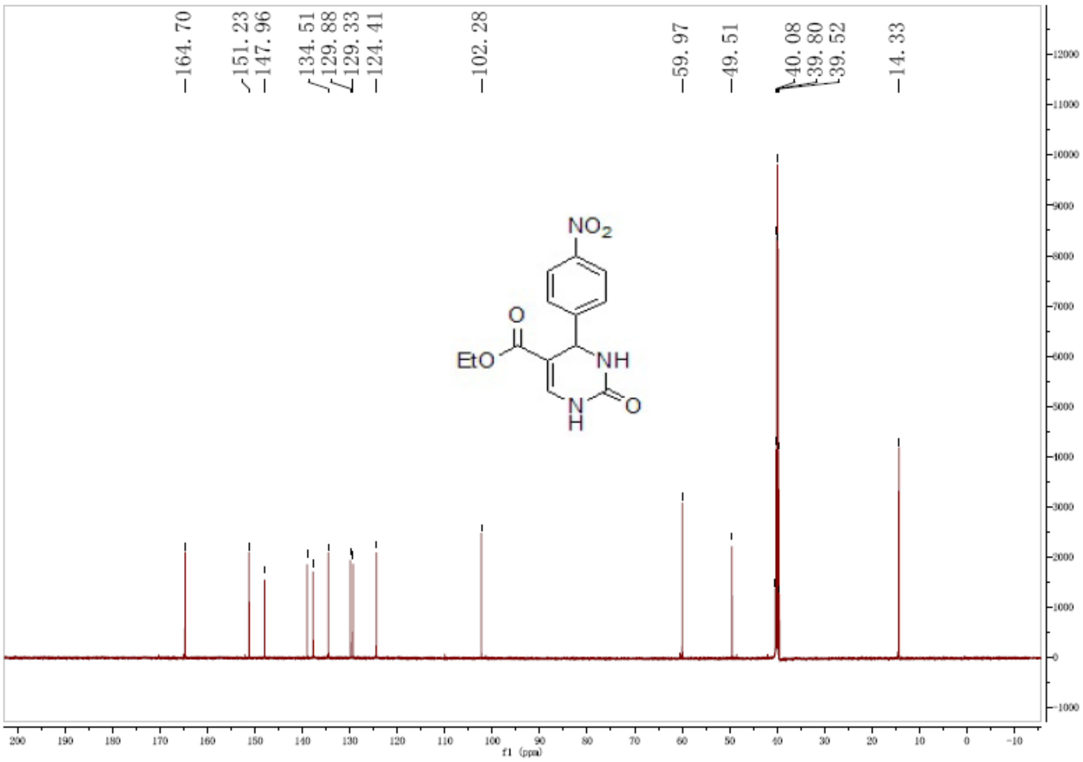

^{1}H and ^{13}C NMR of **5t**

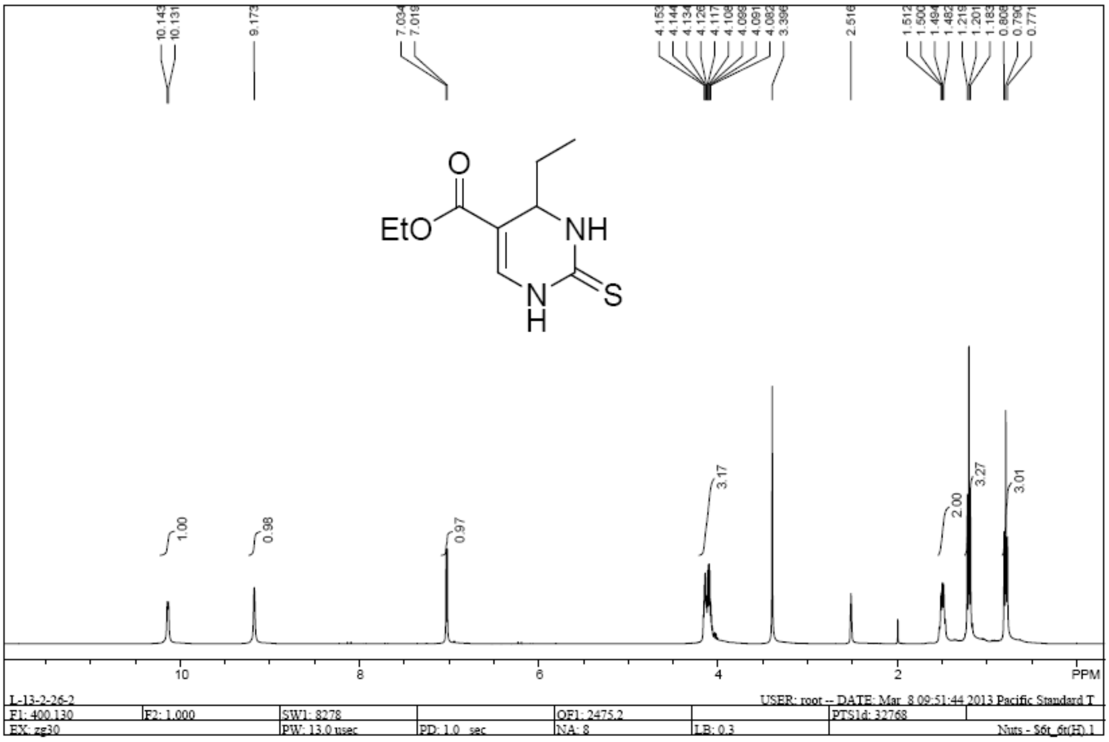

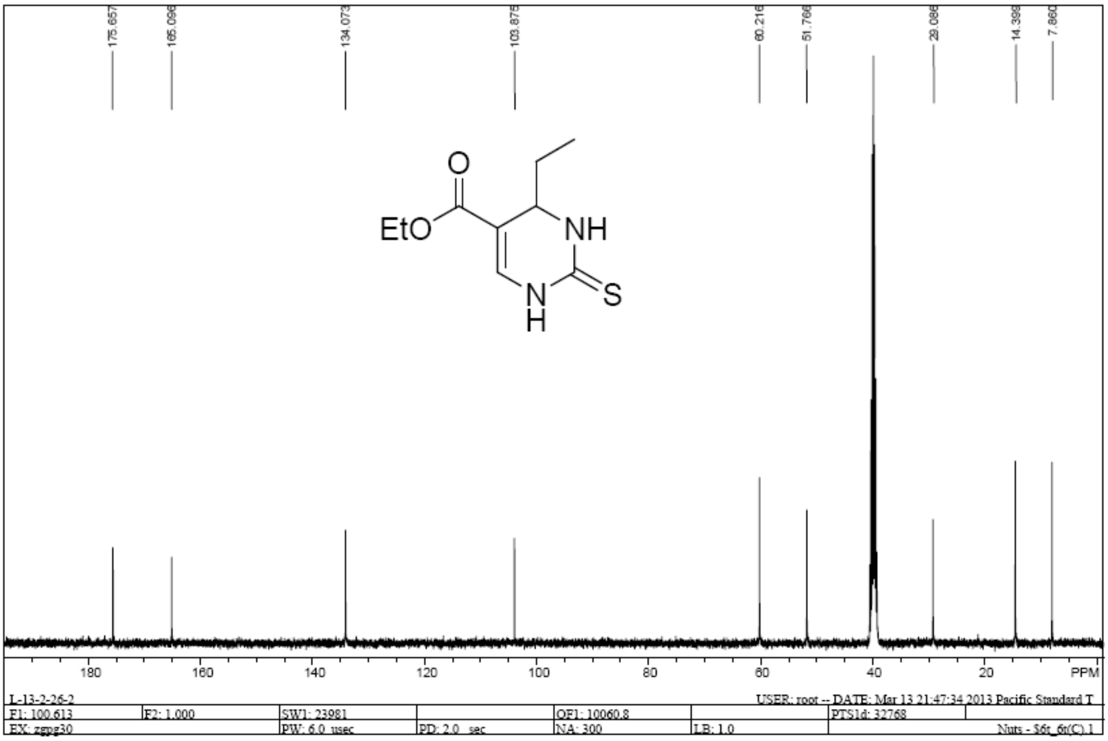

^{1}H and ^{13}C NMR of **5u**

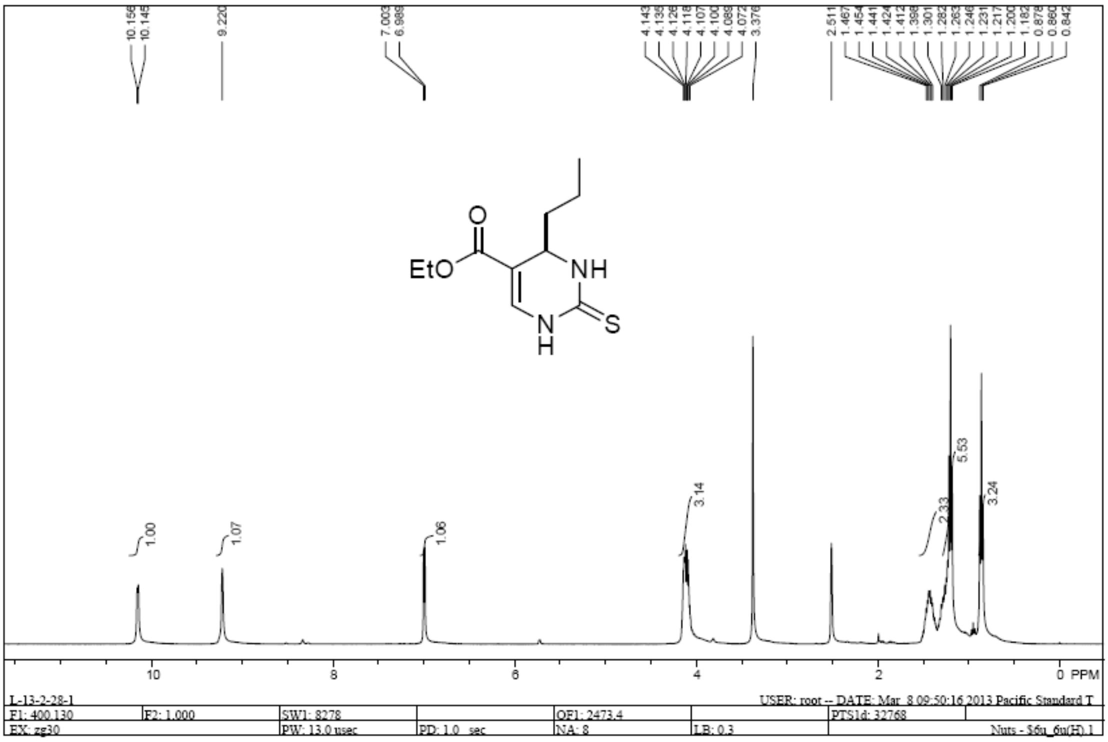

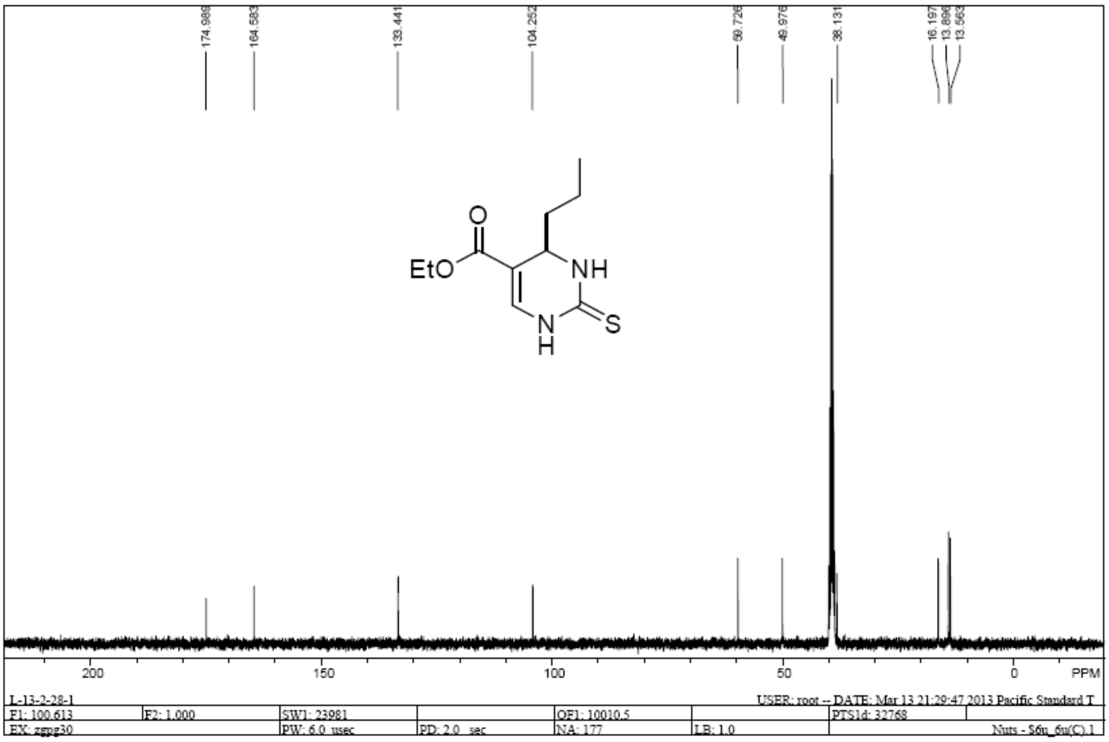

^{1}H and ^{13}C NMR of **5v**

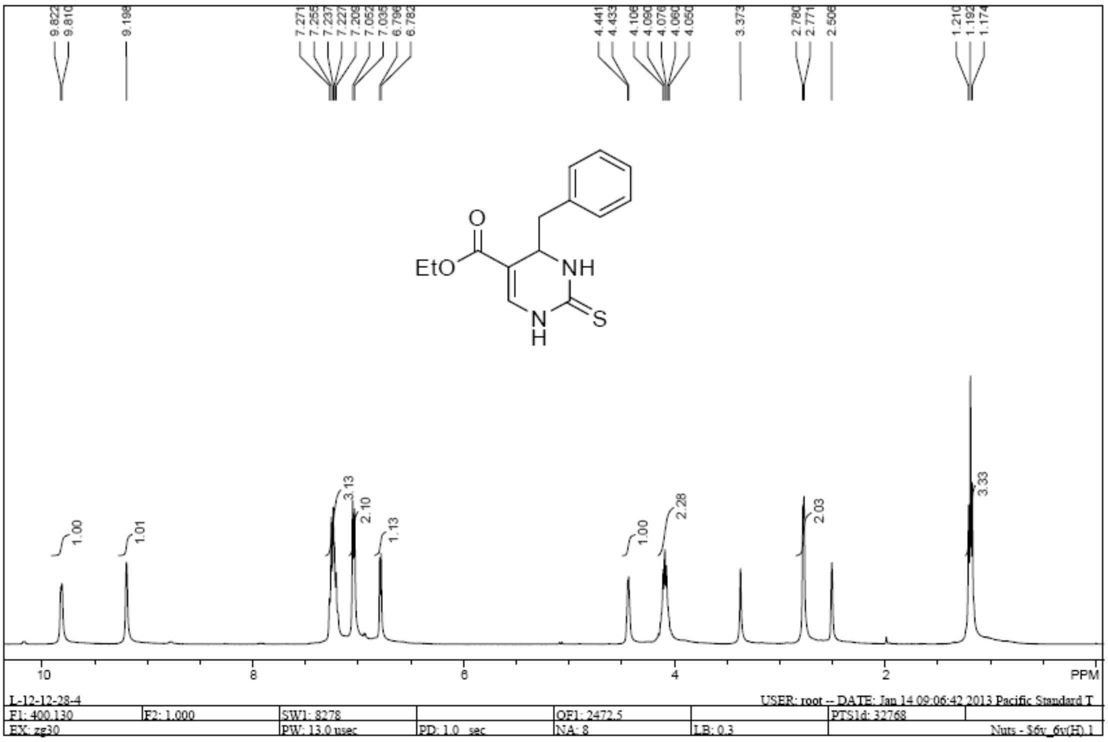

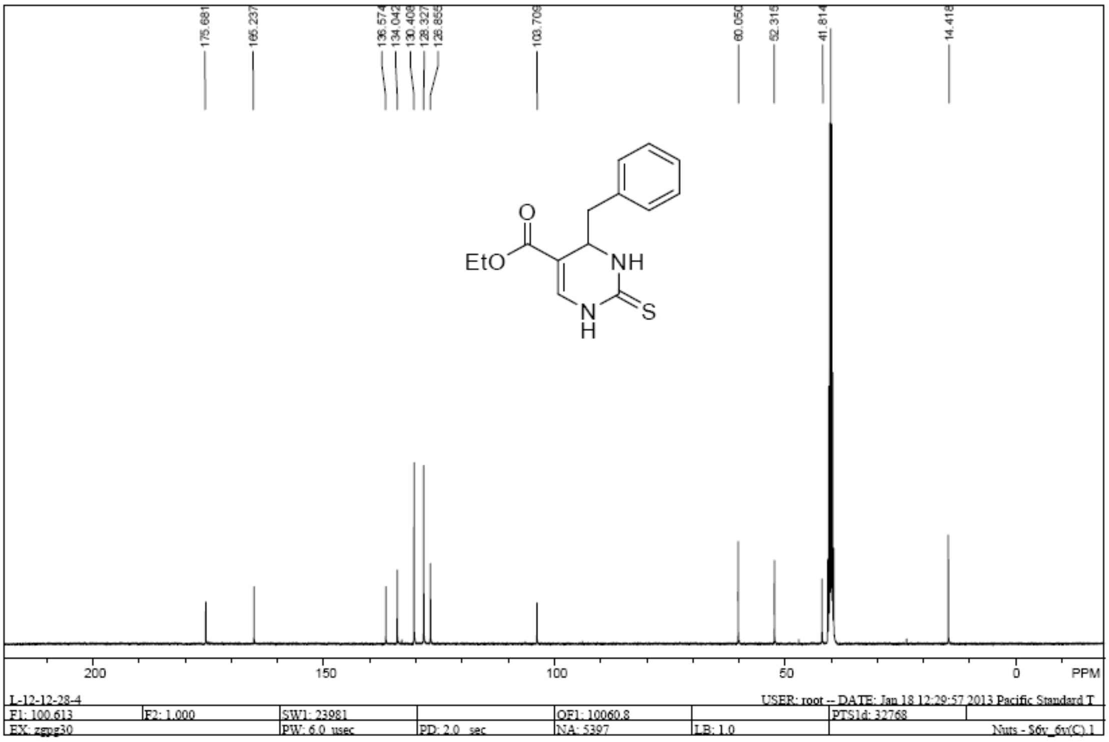

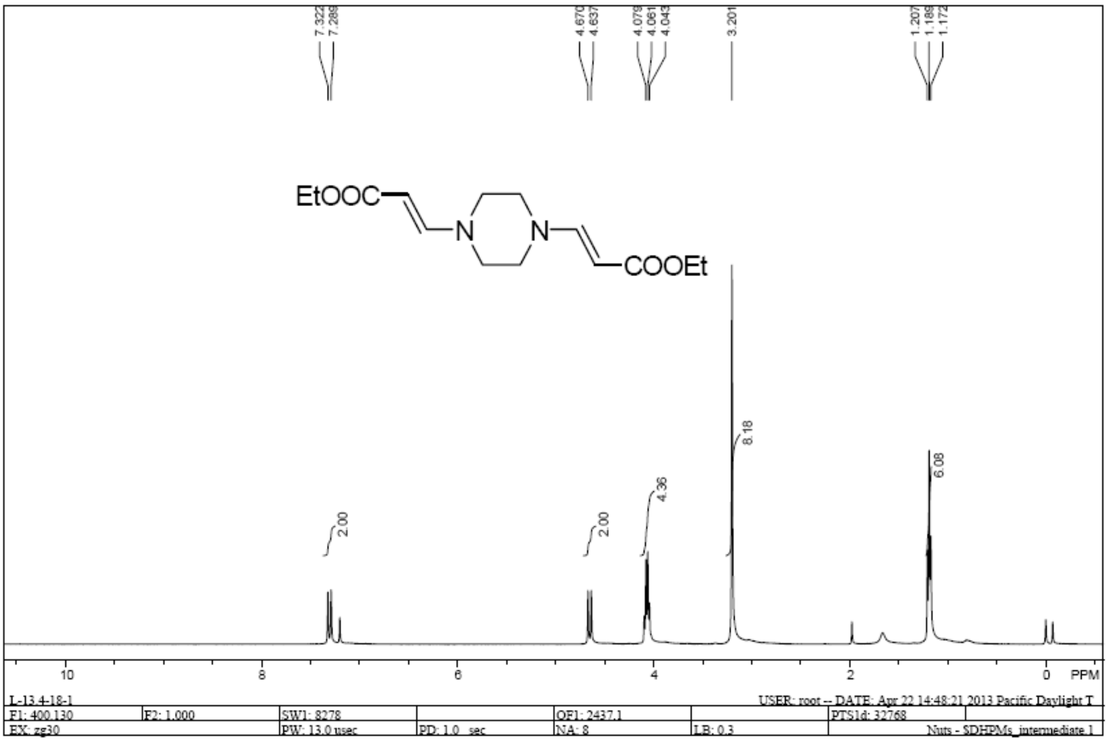
EtOOC
COOEt
N
N
L-13-4-18-1
USER: root -- DATE: Apr 22 14:48:21 2013 Pacific Daylight T
F1: 400.130
F2: 1.000
SW1: 8278
OF1: 2437.1
PTS1d: 32768
EX: zg30
PW: 13.0 usec
PD: 1.0 sec
NA: 8
LB: 0.3
Nuts - $DHPMs_intermediate 1
PPM

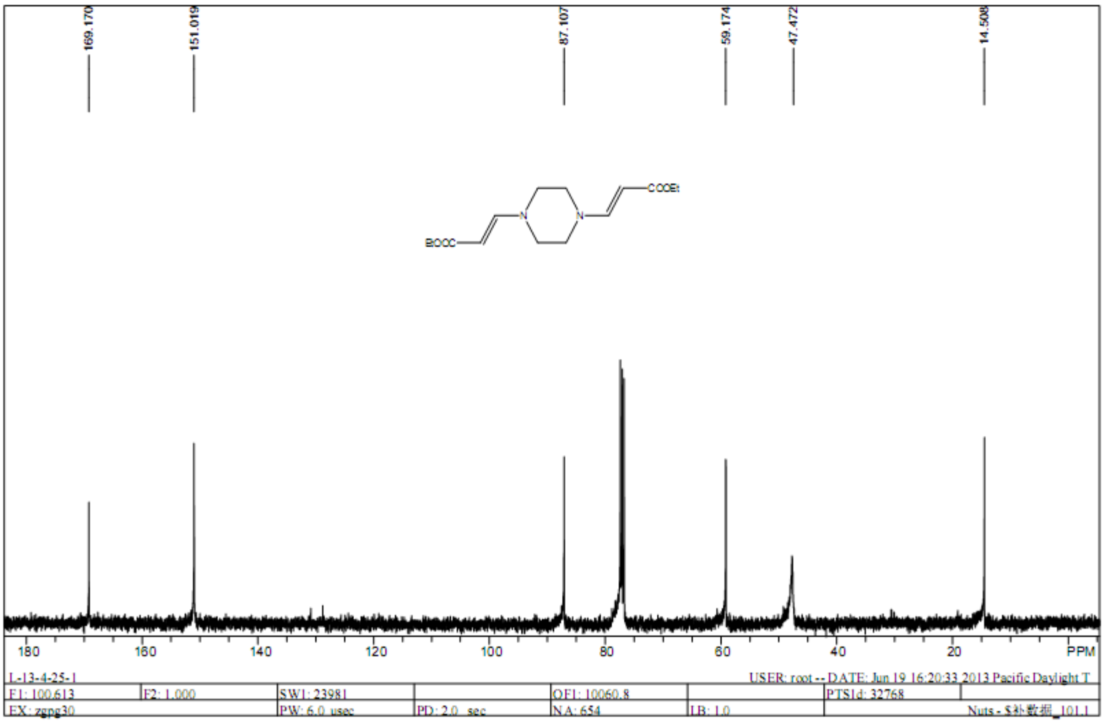
169.170
151.019
87.107
59.174
47.472
14.508
COOEt
EtOOC
L-13-4-25-1
USER: root -- DATE: Jun 19 16:20:33 2013 Pacific Daylight T
F1: 100.613
F2: 1.000
SW1: 23981
OF1: 10060.8
PTS1d: 32768
EX: zgpg30
PW: 6.0 usec
PD: 2.0 sec
NA: 654
LB: 1.0
PPM
